# AI Decision Support for Emergency Triage of Large Vessel Occlusion Using Noncontrast Computed Tomography: Systematic Review and Bayesian Diagnostic Test Accuracy Network Meta-Analysis

**DOI:** 10.2196/105068

**Published:** 2026-09-24

**Authors:** Huiqing Zhou, Li Liu, Bolun Fu, Bin Cao, Jiafu Ma, Kun Yang, Yuzhen Bao, Fengyong Yang, Xin Lian

**Affiliations:** 1 Department of Radiology People's Hospital Affiliated to Shandong First Medical University Jinan, Shandong China; 2 Department of Neurology Jimo District Hospital of Traditional Chinese Medicine Qingdao, Shandong China; 3 Department of Infectious Diseases Qingdao Public Health Clinical Center Qingdao, Shandong China; 4 Department of Emergency Medicine People's Hospital Affiliated to Shandong First Medical University Jinan, Shandong China

**Keywords:** artificial intelligence, AI, large vessel occlusion, noncontrast computed tomography, noncontrast CT, stroke triage, diagnostic test accuracy, network meta-analysis

## Abstract

**Background:**

Emergency triage of anterior circulation large vessel occlusion (LVO) is constrained by delays in vascular imaging, specialist interpretation, and transfer decision-making. Noncontrast computed tomography (NCCT) is often obtained first in suspected stroke, but visual recognition of LVO in NCCT images is difficult outside specialist settings. NCCT-based AI may provide an early human-in-the-loop escalation signal.

**Objective:**

This study aimed to compare validated NCCT-based AI paradigms with human reader paradigms for detection of anterior circulation LVO and assess whether current evidence supports prospective evaluation of AI-assisted escalation pathways.

**Methods:**

We searched the PubMed, Embase, Web of Science Core Collection, and Cochrane Library databases from inception to May 24, 2026. Eligible studies evaluated NCCT-based LVO detection in validation datasets independent of model development, included within-study head-to-head comparisons, and provided reconstructible 2 × 2 data. Four nodes were compared: expert readers, nonexpert readers, unimodal imaging AI, and clinically informed multimodal AI. A Bayesian bivariate hierarchical diagnostic test accuracy network meta-analysis estimated sensitivity, specificity, diagnostic odds ratio, and absolute differences. Risk of bias and applicability were assessed using the Prediction Model Risk of Bias Assessment Tool for AI and the complementary Quality Assessment of Diagnostic Accuracy Studies–3, and certainty was rated using the Grading of Recommendations Assessment, Development, and Evaluation (GRADE).

**Results:**

All 10 included studies were retrospective validation studies, comprising 11 validation datasets, 28 study node arms, and 3632 patients. Unimodal imaging AI had a sensitivity of 0.78 (95% credible interval 0.68-0.86), specificity of 0.88 (95% credible interval 0.81-0.94), and diagnostic odds ratio of 30.89 (95% credible interval 14.19-60.27). Expert readers and nonexpert readers had lower sensitivity estimates of 0.62 (95% credible interval 0.48-0.75) and 0.60 (95% credible interval 0.45-0.75), respectively, with similar specificity estimates of 0.86. In league table comparisons, unimodal imaging AI showed higher sensitivity than expert and nonexpert readers by 0.16 (95% credible interval 0.03-0.29) and 0.18 (95% credible interval 0.03-0.32), respectively, with no clear specificity separation. Clinically informed multimodal AI had a sensitivity of 0.81 (95% credible interval 0.64-0.92) and specificity of 0.92 (95% credible interval 0.80-0.98), but this sparse node was connected to human readers only through indirect evidence.

**Conclusions:**

In retrospective validation cohorts, there was low-certainty comparative evidence suggesting that NCCT-based AI, most clearly unimodal imaging AI, had higher sensitivity than human reader paradigms, without a clear difference in specificity. These findings support prospective evaluation of NCCT-based AI as a bounded human-in-the-loop prompt for expert review, computed tomography angiography prioritization, tele-stroke consultation, or transfer discussion. The evidence remains accuracy based and does not establish workflow effectiveness, reperfusion acceleration, or functional outcome benefit.

**Trial Registration:**

PROSPERO CRD420261362111; https://www.crd.york.ac.uk/PROSPERO/view/CRD420261362111

## Introduction

Anterior circulation large vessel occlusion (LVO) is one of the most disabling and time-sensitive subtypes of acute ischemic stroke (AIS) [1,2]. Endovascular thrombectomy (EVT) improves outcomes, but its benefit declines rapidly with treatment delay. In pooled individual patient data from the HERMES collaboration, each 1-hour delay between onset and reperfusion was associated with a 5.2% absolute reduction in functional independence [3]. Interhospital transfer can add substantial delay; the STRATIS (Systematic Evaluation of Patients Treated With Neurothrombectomy Devices for Acute Ischemic Stroke) registry reported a median treatment delay of 110 minutes and a 7.8–percentage point lower rate of functional independence among transferred patients [4]. Rapid LVO recognition at first medical contact is therefore central to emergency stroke triage.

Computed tomography angiography (CTA) is the first-line noninvasive test for confirming anterior circulation LVO in the emergency setting, whereas digital subtraction angiography (DSA) remains the definitive invasive reference standard [5]. Magnetic resonance angiography (MRA) is used less often in the hyperacute phase because of time and compatibility constraints. Immediate CTA, however, is not consistently available in all hospitals or at all hours, particularly in some resource-constrained or off-hours settings. Barriers include equipment limitations, contrast contraindications, gaps in overnight technologist availability, and logistical constraints in remote settings [6]. When CTA cannot be obtained promptly, noncontrast computed tomography (NCCT) often remains the earliest imaging input for triage. Human detection of anterior circulation LVO in NCCT images is limited, with reported sensitivities of 30% to 52% [7-9], and the hyperdense artery sign has a pooled sensitivity of approximately 52% [10].

NCCT-based AI has therefore been proposed as a decision support layer for emergency LVO triage. Several commercial platforms have been evaluated, including Heuron [11], Brainomix [7,12], RapidAI [8], and Methinks AI [9]. The intended role of NCCT-based AI is not to substitute for CTA but to provide an early signal before vascular imaging confirmation or when CTA is not immediately available. That signal could prompt urgent vascular imaging escalation, tele-stroke consultation, or transfer discussion. Existing reviews have addressed related questions. A recent conventional meta-analysis pooled NCCT-based AI studies for LVO prediction but did not incorporate human reader comparators or a connected evidence network [13]. Another review summarized commercial stroke platforms without quantitative synthesis focused on NCCT-based diagnostic accuracy [14].

For digital stroke triage, the evidentiary question is not only whether NCCT-based AI can detect LVO. The more clinically relevant question is whether the diagnostic signal is robust enough to support escalation decisions in human-in-the-loop workflows before or alongside confirmatory vascular imaging. We therefore conducted a systematic review and Bayesian diagnostic test accuracy network meta-analysis (DTA-NMA) to evaluate whether validated NCCT-based AI provides a comparative diagnostic signal that could justify prospective workflow testing as a bounded escalation aid. This clinical positioning is summarized in Figure 1.

**Figure 1:**
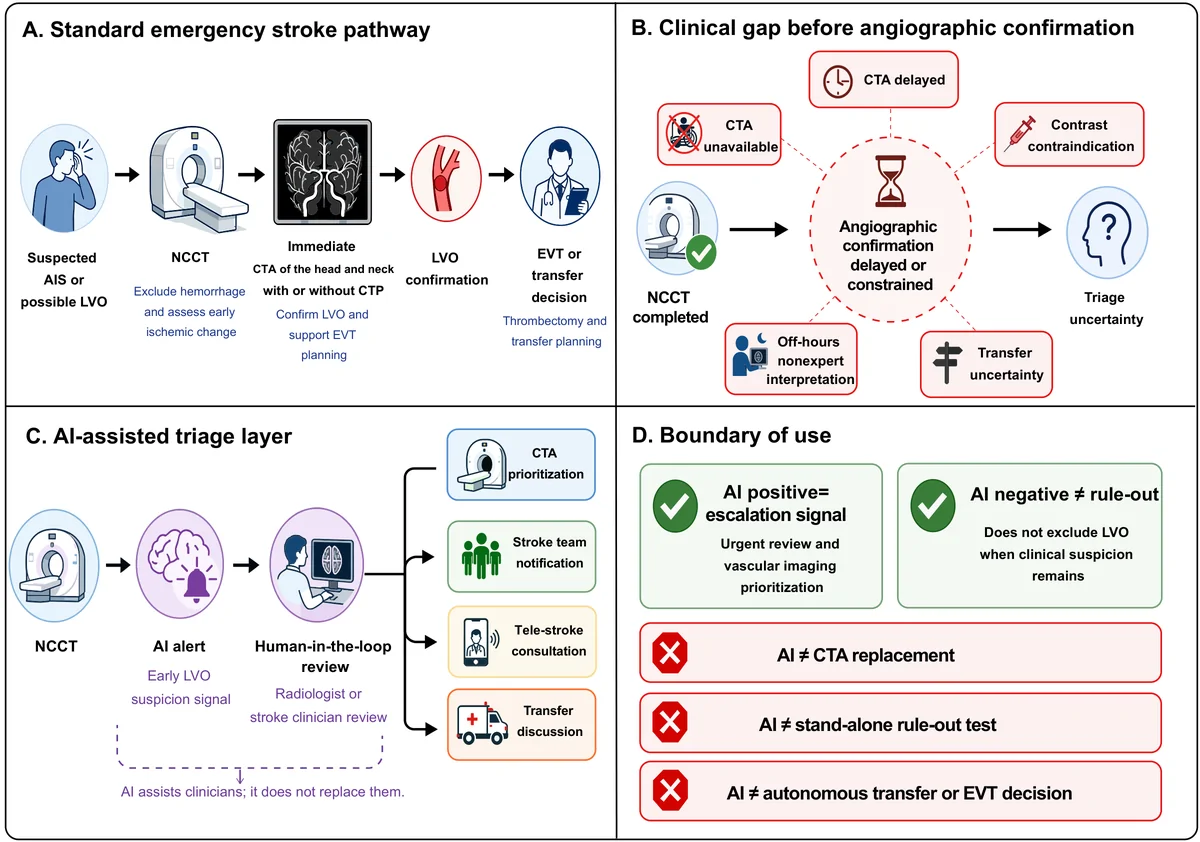
Clinical positioning of NCCT-based AI in emergency anterior circulation LVO triage. AI: artificial intelligence; AIS: acute ischemic stroke; CTA: computed tomography angiography; CTP: computed tomography perfusion; EVT: endovascular thrombectomy; LVO: large vessel occlusion; NCCT: noncontrast computed tomography.

## Methods

### Reporting Guidelines and Protocol Registration

This study followed the PRISMA (Preferred Reporting Items for Systematic Reviews and Meta-Analyses) 2020 statement [15], the PRISMA extension for diagnostic test accuracy (PRISMA-DTA) [16], the PRISMA extension for network meta-analyses (PRISMA-NMA) [17], the PRISMA extension for Abstracts [18], and the PRISMA literature search extension (PRISMA-S) [19]. Completed reporting checklists are provided in Tables S1 to S4 in Multimedia Appendix 1 [15-19]. The protocol was prospectively registered with PROSPERO (CRD420261362111).

### Literature Search Strategy

Two investigators (HZ and LL) systematically searched the PubMed, Embase, Web of Science Core Collection, and Cochrane Library databases from inception to May 24, 2026. The search combined controlled vocabulary and free-text terms across 4 concept blocks: ischemic stroke, occlusion location, NCCT, and AI or automated detection. No restrictions on publication date, language, or study type were applied. Citation tracking of included studies and relevant reviews was also performed. The full reproducible search strategy and final database yields are provided in Table S5 in Multimedia Appendix 1. Two reviewers (HZ and LL) independently screened titles, abstracts, and full texts against the eligibility criteria. Disagreements were resolved through discussion, with adjudication by XL when consensus could not be reached.

### Eligibility Criteria, Validation Standards, and Node Taxonomy

Eligibility criteria were structured using the participants, index tests, target condition, reference standard, outcomes, and study design framework for diagnostic test accuracy reviews [16]. Participants were adults with suspected AIS who underwent baseline NCCT. Index tests comprised 4 mutually exclusive nodes. Expert readers were senior neuroradiologists, stroke specialists, or experienced radiologists explicitly identified as expert or senior readers or reported as having substantial NCCT interpretation experience in the source publications. Nonexpert readers were nonneuroradiology or non–stroke specialist readers, including general radiologists, emergency physicians, junior radiology or neurology residents, or trainees as specified in the source publications. Unimodal imaging AI comprised fully automated algorithms receiving only NCCT images without clinical information. Clinically informed multimodal AI comprised fully automated algorithms integrating NCCT images with immediately available bedside clinical variables such as the National Institutes of Health Stroke Scale (NIHSS) score and time from symptom onset. Variables requiring laboratory turnaround time, such as blood test results, were excluded. The target condition was predominantly anterior circulation LVO, defined as occlusion of the internal carotid artery or middle cerebral artery M1 segment with or without M2 involvement; anterior circulation cases were required to constitute 90% or more of the study cohort.

The reference standard was CTA in the vast majority of included studies; DSA, the definitive angiographic gold standard, and MRA were accepted as alternative reference standards when available. One included cohort used mixed vascular reference standards and a broader reference standard timing window; its influence was evaluated through prespecified sensitivity analysis. Outcomes required sufficient data for complete 2 × 2 contingency table reconstruction. Study design required within-study head-to-head comparisons involving at least 2 of the 4 prespecified diagnostic nodes to ensure clinically meaningful network connectivity. This head-to-head requirement restricted the primary evidence base to studies capable of informing comparative network inference; otherwise relevant single-node validation studies excluded under this criterion are summarized in Table S6 [20-22] in Multimedia Appendix 1. The study by Olive-Gadea et al [23] was retained because anterior circulation occlusions constituted 95% of the validation cohort, satisfying the predefined anterior circulation threshold; its influence was evaluated in sensitivity analysis.

Eligible AI studies had to evaluate diagnostic performance in validation cohorts independent of model development. Acceptable validation designs included independent external validation, independently assembled validation cohorts, and temporal internal validation. Studies relying solely on training set performance, random hold-out splits, cross-validation, or bootstrapping were excluded from the main analysis. Studies were also excluded if they were limited to posterior circulation occlusions; evaluated CTA-only, computed tomography perfusion–only, or magnetic resonance imaging–based AI; lacked sufficient data for 2 × 2 table reconstruction; were single-arm diagnostic studies; were nonoriginal reports; or used overlapping cohorts. These criteria were intended to preserve clinical comparability and support the plausibility of the transitivity assumption; statistical consistency was evaluated separately.

### Data Extraction

Two investigators (JM and FY) independently extracted data using a standardized form; XL adjudicated discrepancies. Extracted information included study design, country, center type, reference standard, validation design, cohort size, LVO prevalence, target vessel segments, NCCT acquisition parameters, AI software or model type, reader category, and 2 × 2 diagnostic data. For studies reporting both internal and external validation, external validation data were preferentially extracted. For multi-reader studies, source-reported group-level results were used when available. Otherwise, reader-level results were averaged within the prespecified expertise category and converted to an integer 2 × 2 table; no noninteger cell counts were entered into the binomial model. Study-specific derivation is detailed in Table S7 in Multimedia Appendix 1 [7-9,11,12,23-27]. Rai et al [7] reported 2 nonoverlapping external validation subsets with different node compositions; these subsets were entered separately because merging them would have disrupted the node-level data structure (Tables S8 and S9 in Multimedia Appendix 1 [7-9,11,12,23-27]). Source-reported reader expertise, comparator structure, and classification basis for expert reader and nonexpert reader nodes are summarized in Table S7 in Multimedia Appendix 1 [7-9,11,12,23-27].

### Quality Assessment and Certainty of Evidence

Two independent reviewers (BF and BC) assessed risk of bias and applicability using the Prediction Model Risk of Bias Assessment Tool for AI (PROBAST+AI) [28]. Diagnostic accuracy risk of bias and applicability were additionally assessed using the Quality Assessment of Diagnostic Accuracy Studies–3 (QUADAS-3) across the participants, index test, target condition, and analysis domains [29]. Discrepancies were resolved by XL. The Grading of Recommendations Assessment, Development, and Evaluation (GRADE) framework was used to rate certainty for node-specific and major comparative sensitivity and specificity estimates [30]. Risk-of-bias judgments within the GRADE framework were informed by the PROBAST+AI and QUADAS-3 assessments. Domain-level judgments and evidence profiles are provided in Tables S10 to S12 [7-9,11,12,23-29], S13 [30], and S14 [7-9,11,12,23-30] in Multimedia Appendix 1.

### Diagnostic Accuracy Measures

Sensitivity quantified correct identification of anterior circulation LVO, and specificity quantified correct exclusion of LVO. Because emergency LVO triage is primarily intended to reduce missed occlusions, sensitivity and false negative consequences were prespecified as the primary clinically relevant dimensions. Specificity and false positive transfer burden were interpreted as secondary clinical dimensions. The diagnostic odds ratio (DOR) and surface under the cumulative ranking curve (SUCRA) were treated as global, exploratory summaries rather than the primary basis for clinical ranking [31].

### Statistical Analysis

The Bayesian DTA-NMA was performed in R (version 4.5.2; R Foundation for Statistical Computing) using *R2jags*. A bivariate hierarchical random-effects model jointly estimated sensitivity and specificity while accounting for potential threshold effects [32,33]. For cohorts contributing more than one diagnostic node, rows were indexed using a shared cohort identifier and modeled using common cohort-level random effects together with cohort-by-node relative random effects, thereby accounting for clustering of diagnostic arms within the same validation cohort. Weakly informative normal priors (mean 0; precision=0.01 [variance=100]) were assigned to logit-transformed sensitivity and specificity parameters. In the primary model, uniform(0, 2) priors were assigned to the between-study SDs, and a uniform(−0.99, 0.99) prior was assigned to the correlation coefficient ρ. Posterior distributions were estimated using Markov chain Monte Carlo (MCMC) simulation with 4 chains, 10,000 burn-in iterations, and 200,000 sampling iterations per chain; chains were thinned every 10 iterations, yielding 80,000 retained posterior draws for inference. Convergence was assessed via trace plot inspection and the potential scale reduction factor, with values below 1.05 considered acceptable [34].

Between-study heterogeneity was quantified using τ on the logit scale [35]. Local inconsistency was assessed through node splitting when an independent indirect comparison was identifiable. Global inconsistency was assessed using the design-by-treatment interaction model, with model fit compared using the deviance information criterion (DIC); ΔDIC was defined as DIC for the inconsistency model minus DIC for the consistency model [36]. SUCRA values were calculated as descriptive summaries of diagnostic hierarchy rather than definitive evidence of clinical superiority [37]. League tables of relative DORs (rDORs) and absolute differences in sensitivity and specificity were generated; the rDOR league table is provided in Table S15 in Multimedia Appendix 1. Clinical utility was evaluated using absolute effects per 1000 patients and Fagan nomograms at pretest probabilities of 20%, 30%, and 49.8% [38]. The 20% scenario was used as the primary clinical illustration for suspected AIS or emergency triage populations, 30% represented confirmed or high-suspicion AIS populations [39,40], and 49.8% represented the enriched median prevalence of the included validation cohorts.

Meta-regression explored 4 covariates: NCCT slice thickness (<3 vs ≥3 mm), M2 occlusion inclusion, validation strategy, and algorithm origin (commercial vs laboratory developed). The validation strategy and algorithm origin covariates were restricted to AI nodes because internal vs external model validation and algorithm development origin do not apply to human visual assessment. Five data structure sensitivity analyses were performed. Sensitivity analysis 1 restricted the network to independent external validation and anterior circulation cohorts and excluded the study by Olive-Gadea et al [23]. Sensitivity analysis 2 excluded the sole laboratory-developed AI model reported by Tolhuisen et al [24]. Sensitivity analysis 3 retained M2-including studies only. Sensitivity analysis 4 removed subset A in the study by Rai et al [7]. Sensitivity analysis 5 excluded the US cohort in the study by Sunwoo et al [25]. These analyses evaluated robustness to anterior circulation restriction, validation design, algorithm origin, M2 inclusion, dual subset handling, and mixed or nonconcurrent reference standard characteristics. Small-study effects were assessed using the Deeks funnel plot asymmetry test [41].

To assess robustness to heterogeneity prior assumptions, an additional prior sensitivity analysis (sensitivity analysis 6) refitted the model after replacing the primary uniform(0, 2) priors for the between-study SDs with weakly informative half-normal(0, 1) priors [42]. The likelihood, node definitions, correlation prior, MCMC settings, convergence criteria, and diagnostic data structure were otherwise unchanged. Sensitivity analysis 6 results were compared with those of the primary model for node-level sensitivity, specificity, and DOR and the exploratory diagnostic hierarchy.

Transitivity was assessed by comparing key potential effect modifiers across studies and nodes, including reader expertise, target vessel definition, M2 inclusion, NCCT section thickness, reference standard, LVO prevalence, validation design, algorithm type, and multimodal clinical inputs (Table S16 in Multimedia Appendix 1).

### Ethical Considerations

This study used published aggregate data and did not involve new individual-level patient data. Additional institutional review board approval or informed consent was therefore not required.

## Results

### Literature Search and Study Selection

Database searches up to May 24, 2026, identified 1775 records (PubMed: n=393, 22.1%; Embase: n=860, 48.5%; Web of Science: n=486, 27.4%; Cochrane Library: n=36, 2%). After deduplication and screening, 10 independent studies [7-9,11,12,23-27] encompassing 11 validation cohorts, 28 study node arms, and 3632 patients met the eligibility criteria (Figure 2A). Reasons for exclusion of the single-arm studies are detailed in Table S6 [20-22] in Multimedia Appendix 1.

**Figure 2:**
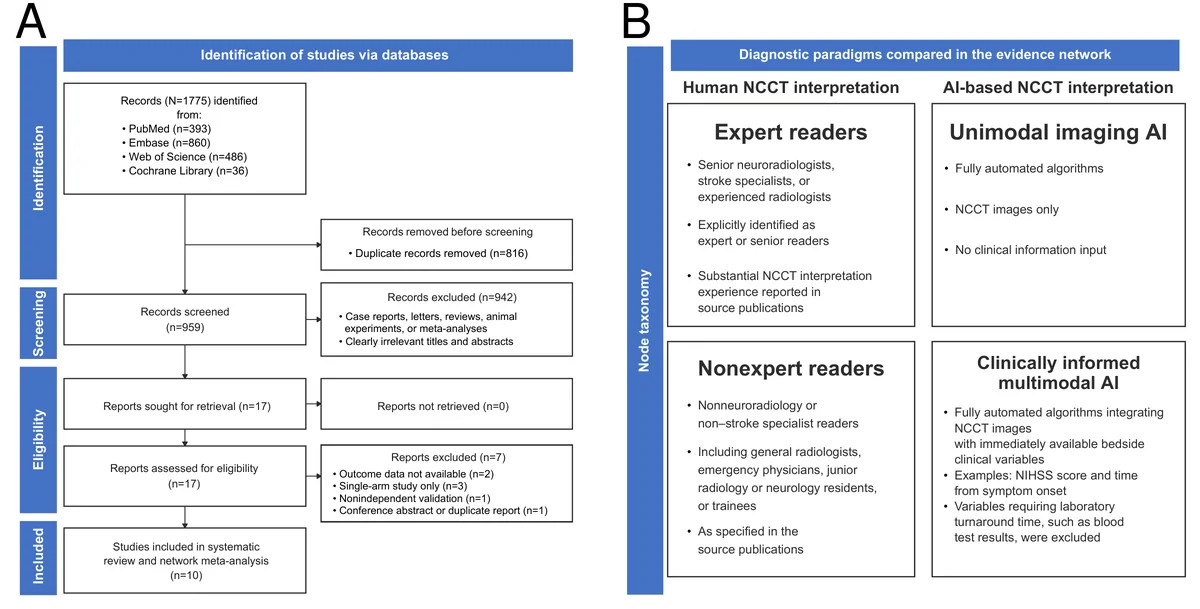
Study selection and diagnostic node taxonomy: (A) PRISMA (Preferred Reporting Items for Systematic Reviews and Meta-Analyses) flow diagram showing record identification, screening, eligibility assessment, and inclusion; and (B) definitions of the 4 mutually exclusive diagnostic nodes compared in the Bayesian diagnostic test accuracy network meta-analysis. NCCT: noncontrast computed tomography; NIHSS: National Institutes of Health Stroke Scale.

### Study Characteristics and Validation Profile

The 10 included studies were published between 2020 and 2026 and were conducted in South Korea, the United States, Spain, the United Kingdom, Germany, Greece, Chile, Brazil, and the Netherlands. Baseline characteristics are summarized in Table 1, with additional validation cohort demographics and NCCT acquisition details provided in Tables S8 and S9 in Multimedia Appendix 1 [7-9,11,12,23-27]. All included studies were retrospective validation studies. In total, 90% (9/10) of the studies evaluated AI software developed by commercial companies, whereas 10% (1/10) of the studies evaluated a laboratory-developed AI model. A total of 90% (9/10) of the studies used independent external validation, and 10% (1/10) used temporal internal validation. CTA was the reference standard in 90% (9/10) of the studies, comprising 3392 participants; 10% (1/10) of the studies, comprising 240 participants, used mixed CTA, MRA, and DSA reference standards. Potential effect modifiers varied across studies, particularly in reader expertise, M2 inclusion, NCCT section thickness, LVO prevalence, and multimodal clinical inputs (Table S16 in Multimedia Appendix 1).

**Table 1:** Baseline characteristics of the included studies^a^.

Studies	Nodes^b^	Design	Center	Source	Country	Reference standard	Training set (LVO^c^/total)	Internal validation (LVO/total)	External validation (LVO/total)
Lee et al [11], 2025	1, 2, and 3	Retrospective	Single center	Local hospital	South Korea	CTA^d^	NR^e^	NR	112/477
Rai et al [7], 2025	1, 2, 3, and 4	Retrospective	Multicenter	Local hospital	United States	CTA	NR	NR	304/612
Yedavalli et al [8], 2023	1, 2, and 3	Retrospective	Multicenter	CRISP^f^ and DEFUSE 3^g^	United States, Chile, and Brazil	CTA	NR	NR	115/244
Weyland et al [12], 2022	2 and 3	Retrospective	Multicenter	Local hospital	Germany, Greece, and United Kingdom	CTA	275/500	NR	84/154
Chung et al [26], 2025	1 and 3	Retrospective	Multicenter	Local hospital	South Korea	CTA	NR	NR	35/65
Urra et al [9], 2025	1, 2, and 3	Retrospective	Multicenter	Local hospital	United States and Spain	CTA	NR	NR	71/189
Olive-Gadea et al [23], 2020	3 and 4	Retrospective	Multicenter	Local hospital	Spain	CTA	NR/24214	823/1453	NR
Kim et al [27], 2024	3 and 4	Retrospective	Multicenter	Local hospital	South Korea	CTA	419/2463	45/275	25/95
Tolhuisen et al [24], 2020	1, 2, and 3	Retrospective	Multicenter	MR CLEAN^h^	The Netherlands	CTA	86/86	43/43	58/103
Sunwoo et al [25], 2026 (US cohort)	1, 2, and 3	Retrospective	NR	Segmed	United States	CTA, MRA^i^, and DSA^j^	NR	NR	120/240

^a^The study by Rai et al [7] included 2 nonoverlapping external validation subsets; this table reports them as a combined baseline validation cohort, whereas diagnostic analyses entered subsets A and B separately. The study by Olive-Gadea et al [23] contributed a temporal internal validation cohort independent of model development and was excluded in sensitivity analysis 1. Sunwoo et al [25] contributed a Segmed-derived US external validation cohort with mixed reference standards (computed tomography angiography, magnetic resonance angiography, and digital subtraction angiography) and a reference standard window within 24 hours; this cohort was excluded in sensitivity analysis 5. Only validation data independent of model development contributed to the Bayesian diagnostic test accuracy network meta-analysis.

^b^Node 1: expert readers; node 2: nonexpert readers; node 3: unimodal imaging AI; node 4: clinically informed multimodal AI.

^c^LVO: large vessel occlusion.

^d^CTA: computed tomography angiography.

^e^NR: not reported.

^f^CRISP: Computed Tomographic Perfusion to Predict Response to Recanalization in Ischemic Stroke.

^g^DEFUSE 3: Diffusion and Perfusion Imaging Evaluation for Understanding Stroke Evolution 3.

^h^MR CLEAN: Multicenter Randomized Clinical Trial of Endovascular Treatment for Acute Ischemic Stroke in the Netherlands.

^i^MRA: magnetic resonance angiography.

^j^DSA: digital subtraction angiography.

### Full 2 × 2 Diagnostic Data and Node Composition

Complete 2 × 2 diagnostic data for all 28 study node arms are shown in Table 2. Expert readers were predominantly senior neuroradiologists, stroke specialists, or experienced radiologists, whereas nonexpert readers included general radiologists, emergency physicians, and junior radiology or neurology trainees, as reported in the source studies. These data formed the basis of the Bayesian DTA-NMA and enabled comparison across the 4 predefined diagnostic paradigms (Figure 2B). Node distribution was *k*=7 for expert readers, *k*=7 for nonexpert readers, *k*=11 for unimodal imaging AI, and *k*=3 for clinically informed multimodal AI. Individual study-level forest plots for sensitivity, specificity, and DOR are provided in Figures S1 to S4 in Multimedia Appendix 1.

**Table 2:** Diagnostic performance of the included models^a^.

Studies and nodes^b^		Reader group or AI model	Input data modality	Clinical variables	Validation design	TPs^c^, n	FPs^d^, n	FNs^e^, n	TNs^f^, n	Sensitivity	Specificity
**Lee et al [11], 2025**											
	1	Expert readers	Pure image (NCCT^g^)	None	External	85	62	27	303	0.76	0.83
	2	Nonexpert readers	Pure image (NCCT)	None	External	75	118	37	247	0.67	0.68
	3	Heuron ELVO	Pure image (NCCT)	None	External	99	32	13	333	0.88	0.91
**Rai et al [7], 2025**											
	1—subset A	Expert readers	Pure image (NCCT)	None	External	55	6	58	108	0.49	0.95
	2—subset A	Nonexpert readers	Pure image (NCCT)	None	External	53	17	60	97	0.47	0.85
	3—subset A	Brainomix 360 Stroke	Pure image (NCCT)	None	External	78	11	35	103	0.69	0.90
	3—subset B	Brainomix 360 Stroke	Pure image (NCCT)	None	External	126	12	65	182	0.66	0.94
	4—subset B	Brainomix 360 Stroke+NIHSS^h^	Multimodal (NCCT+clinical)	NIHSS	External	124	2	67	192	0.65	0.99
**Yedavalli et al [8], 2023**											
	1	Expert readers	Pure image (NCCT)	None	External	59	17	56	112	0.51	0.87
	2	Nonexpert readers	Pure image (NCCT)	None	External	47	4	68	125	0.41	0.97
	3	Rapid NCCT Stroke (RapidAI)	Pure image (NCCT)	None	External	73	6	42	123	0.63	0.95
**Weyland et al [12], 2022**											
	2	Nonexpert readers	Pure image (NCCT)	None	External	73	11	11	59	0.87	0.84
	3	Brainomix 360 Stroke	Pure image (NCCT)	None	External	65	9	19	61	0.77	0.87
**Chung et al [26], 2025**											
	1	Expert readers	Pure image (NCCT)	None	External	19	4	16	26	0.54	0.87
	3	JLK CTL (JLK)	Pure image (NCCT)	None	External	27	2	8	28	0.77	0.93
**Urra et al [9], 2025**											
	1	Expert readers	Pure image (NCCT)	None	External	30	8	41	110	0.42	0.93
	2	Nonexpert readers	Pure image (NCCT)	None	External	21	13	50	105	0.30	0.89
	3	Methinks LVO (Methinks AI)	Pure image (NCCT)	None	External	58	13	13	105	0.82	0.89
**Olive-Gadea et al [23], 2020**											
	3	Methinks LVO (Methinks AI)	Pure image (NCCT)	None	Internal (temporal)	685	181	138	449	0.83	0.71
	4	Methinks LVO+ (Methinks)	Multimodal (NCCT+clinical)	NIHSS and time from onset	Internal (temporal)	684	94	139	536	0.83	0.85
**Kim et al [27], 2024**											
	3	JLK CTL (JLK)	Pure image (NCCT)	None	External	20	8	5	62	0.80	0.89
	4	JLK CTL+ (JLK)	Multimodal (NCCT+clinical)	NIHSS	External	23	13	2	57	0.92	0.81
**Tolhuisen et al [24], 2020**											
	1	Expert readers	Pure image (NCCT)	None	External	54	18	4	27	0.93	0.60
	2	Nonexpert readers	Pure image (NCCT)	None	External	45	5	13	40	0.78	0.89
	3	3D CNN^i^ (laboratory developed)	Pure image (NCCT)	None	External	50	14	8	31	0.86	0.69
**Sunwoo et al [25], 2026 (US cohort)**											
	1	Expert readers	Pure image (NCCT)	None	External	63	16	57	104	0.53	0.87
	2	Nonexpert readers	Pure image (NCCT)	None	External	76	24	44	96	0.63	0.80
	3	JLK CTL (JLK)	Pure image (NCCT)	None	External	95	8	25	112	0.79	0.93

^a^Study node arms represent unique combinations of validation cohort and diagnostic paradigm. For multi-reader studies, source-reported group-level performance was used when available; otherwise, reader-level results were averaged within the prespecified reader category and reconstructed as integer 2 × 2 counts. Detailed derivation is provided in Table S7 [7-9,11,12,23-27] in Multimedia Appendix 1. Laboratory-developed AI denotes a noncommercial algorithm developed by an academic or research group without commercial company involvement. In this review, only the study by Tolhuisen et al [24] met this criterion. All other AI algorithms were developed by commercial companies (Heuron, Brainomix, RapidAI, Methinks AI, and JLK) regardless of whether regulatory clearance had been obtained at the time of data collection.

^b^Node 1: expert readers; node 2: nonexpert readers; node 3: unimodal imaging AI (pure noncontrast computed tomography input); node 4: clinically informed multimodal AI (noncontrast computed tomography plus clinical variables).

^c^TP: true positive.

^d^FP: false positive.

^e^FN: false negative.

^f^TN: true negative.

^g^NCCT: noncontrast computed tomography.

^h^NIHSS: National Institutes of Health Stroke Scale.

^i^CNN: convolutional neural network.

### Network Geometry, Model Convergence, and Consistency

The evidence network was connected across the 4 diagnostic paradigms, with edge thickness proportional to the volume of within-study head-to-head comparisons (Figure 3). The clinically informed multimodal AI node was connected to human-reader nodes only through the unimodal imaging AI node; comparisons with human readers were therefore entirely indirect. MCMC diagnostics showed satisfactory convergence for node-level sensitivity, specificity, and DOR estimates, with adequate trace plot mixing, stable posterior density overlays, and potential scale reduction factor values below 1.05. The design-by-treatment interaction model yielded a ΔDIC of −0.19, representing negligible separation in model fit between the inconsistency and consistency models. No compelling global inconsistency signal was identified, although node splitting suggested potential local inconsistency for sensitivity between expert and nonexpert readers and the sparse network limited the power to detect inconsistency. Detailed direct and indirect evidence structures and posterior inconsistency factors are provided in Table S17 in Multimedia Appendix 1. Convergence and consistency diagnostics are shown in Figures S5 to S8 in Multimedia Appendix 1.

**Figure 3:**
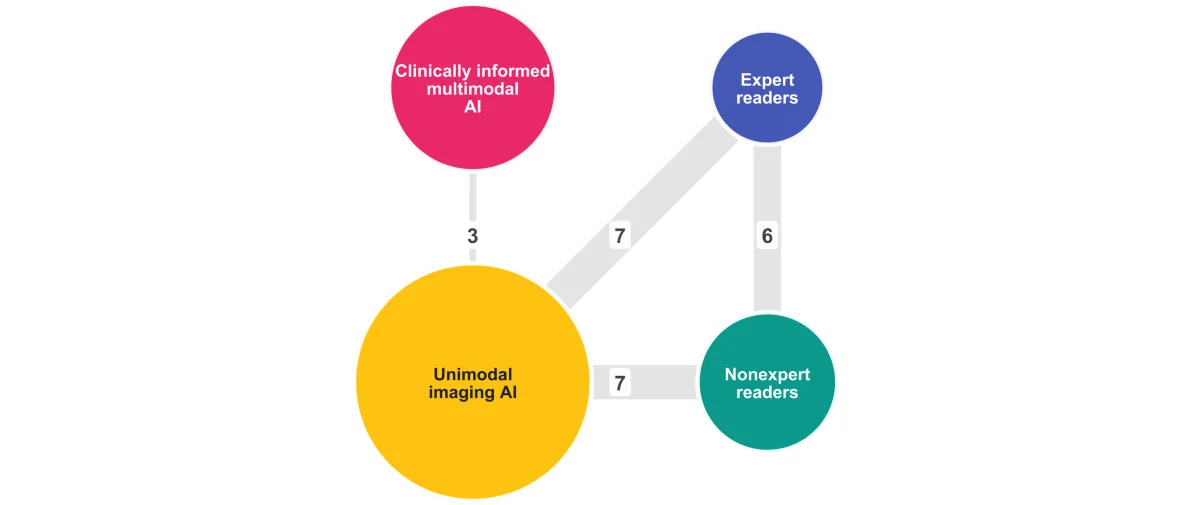
Network geometry of the 4 noncontrast computed tomography (NCCT)–based diagnostic paradigms for detecting anterior circulation large vessel occlusion. Node area is proportional to cumulative patient sample size; edge thickness and superimposed numbers indicate the volume of direct head-to-head comparisons. Node 1: expert readers; node 2: nonexpert readers; node 3: unimodal imaging AI (pure NCCT input); node 4: clinically informed multimodal AI (NCCT plus clinical variables).

### Risk of Bias and Certainty of Evidence

Validation-stage PROBAST+AI assessments were low risk in 90% (9/10) of the studies and unclear in 10% (1/10), whereas complementary QUADAS-3 assessments identified high risk in the participants domain in all studies (10/10, 100%) and high participant applicability concerns in 90% (9/10) of the studies. Additional study-specific concerns were identified in the index test, target condition, and analysis domains; overall QUADAS-3 risk of bias and overall applicability concerns were high in all studies (10/10, 100%; Figure 4; Tables S10-S12 [7-9,11,12,23-29] and Figure S9 [29] in Multimedia Appendix 1). GRADE certainty was low for 7 of 8 node-specific estimates and moderate for unimodal imaging AI specificity. Comparative certainty was low for unimodal imaging AI vs either reader node and for clinically informed multimodal AI vs unimodal imaging AI and very low for clinically informed multimodal AI vs either reader node (Tables S13 [30] and S14 [7-9,11,12,23-30] in Multimedia Appendix 1).

**Figure 4:**
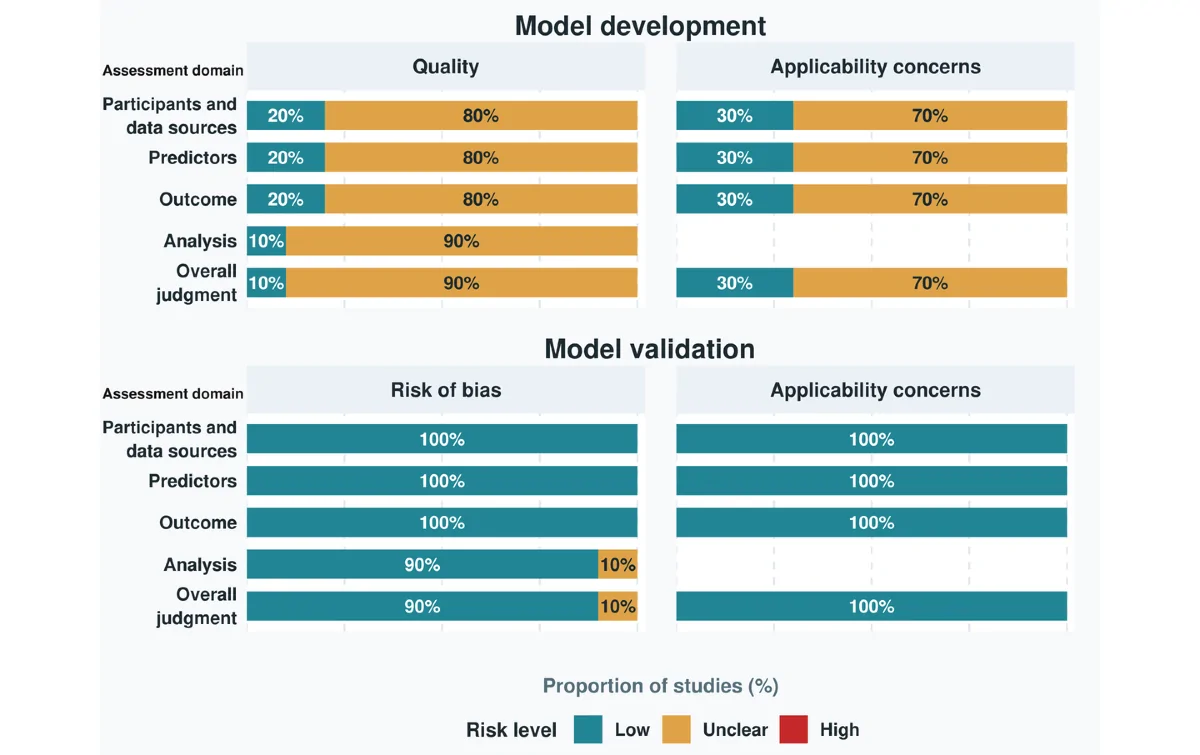
Risk-of-bias and applicability assessment using the Prediction Model Risk of Bias Assessment Tool for AI for the 10 included studies.

### Primary Diagnostic Performance: Sensitivity, Specificity, and Absolute Effects

Under the prespecified clinical interpretation framework, sensitivity and false negative burden were treated as the primary dimensions for emergency LVO triage, whereas specificity and false positive transfer burden were treated as secondary dimensions. Pooled sensitivity and specificity estimates are summarized in Figure 5 and Figure 6 and in Figures S10 and S11 in Multimedia Appendix 1. Clinically informed multimodal AI had a sensitivity of 0.81 with a 95% credible interval of 0.64 to 0.92 and specificity of 0.92 (95% credible interval 0.80-0.98). Unimodal imaging AI had a sensitivity of 0.78 (95% credible interval 0.68-0.86) and specificity of 0.88 (95% credible interval 0.81-0.94). Expert readers had a sensitivity of 0.62 (95% credible interval 0.48-0.75) and specificity of 0.86 (95% credible interval 0.77-0.93). Nonexpert readers had a sensitivity of 0.60 (95% credible interval 0.45-0.75) and specificity of 0.86 (95% credible interval 0.75-0.93). These estimates indicated a clearer signal for sensitivity than for specificity.

**Figure 5:**
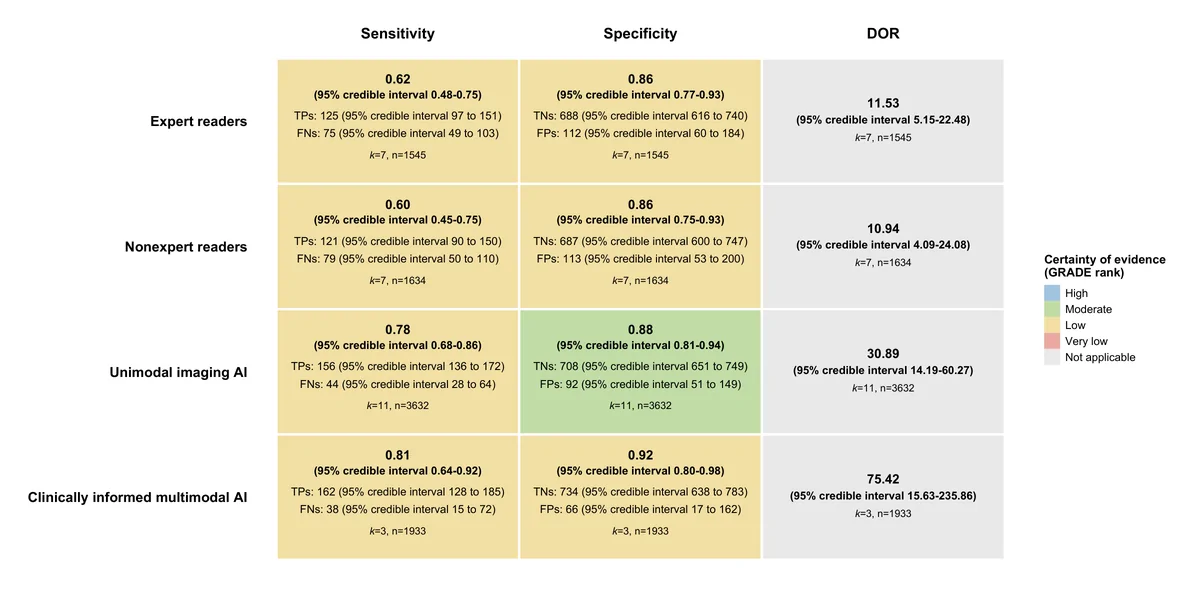
Pooled diagnostic accuracy, certainty of evidence, and absolute diagnostic outcomes per 1000 patients at a 20% pretest probability. DOR: diagnostic odds ratio; FN: false negative; FP: false positive; TN: true negative; TP: true positive.

**Figure 6:**
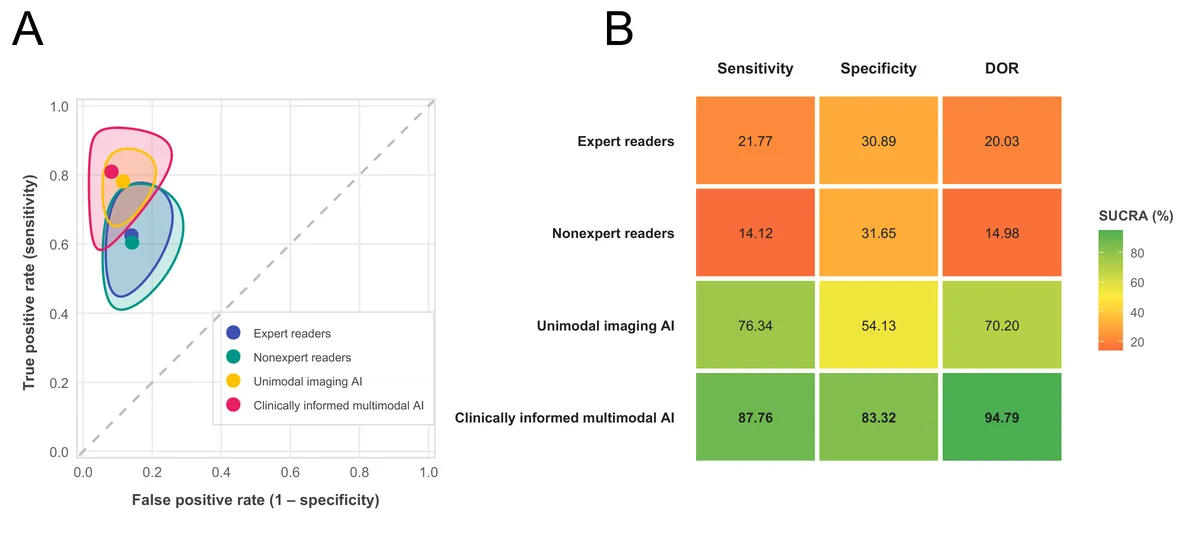
Diagnostic performance across four NCCT-based paradigms: (A) pooled sensitivity and specificity with 95% credible regions; (B) SUCRA values for sensitivity, specificity, and diagnostic odds ratio. DOR: diagnostic odds ratio; SUCRA: surface under the cumulative ranking curve.

### Relative Diagnostic Accuracy and Exploratory Rankings

Exploratory DOR estimates followed the same broad pattern: clinically informed multimodal AI had a DOR of 75.42 (95% credible interval 15.63-235.86), unimodal imaging AI had a DOR of 30.89 (95% credible interval 14.19-60.27), expert readers had a DOR of 11.53 (95% credible interval 5.15-22.48), and nonexpert readers had a DOR of 10.94 (95% credible interval 4.09-24.08; Figure 5; Figure S12 in Multimedia Appendix 1).

The rDOR league table showed higher DOR point estimates for AI diagnostic nodes than for human reader nodes, but these comparisons were interpreted as exploratory summaries rather than direct clinical rankings (Table S15 in Multimedia Appendix 1). The clearest relative sensitivity signal was observed for unimodal imaging AI, which showed higher sensitivity than expert readers and nonexpert readers by 0.16 (95% credible interval 0.03-0.29) and 0.18 (95% credible interval 0.03-0.32), respectively (Figure 7). Clinically informed multimodal AI had higher sensitivity point estimates than human reader nodes, but the corresponding credible intervals were wide and crossed or touched 0. Its rDOR comparison with unimodal imaging AI was also inconclusive at 2.04 (95% credible interval 0.55-7.83). All specificity differences had 95% credible intervals crossing 0 (Figure 7).

**Figure 7:**
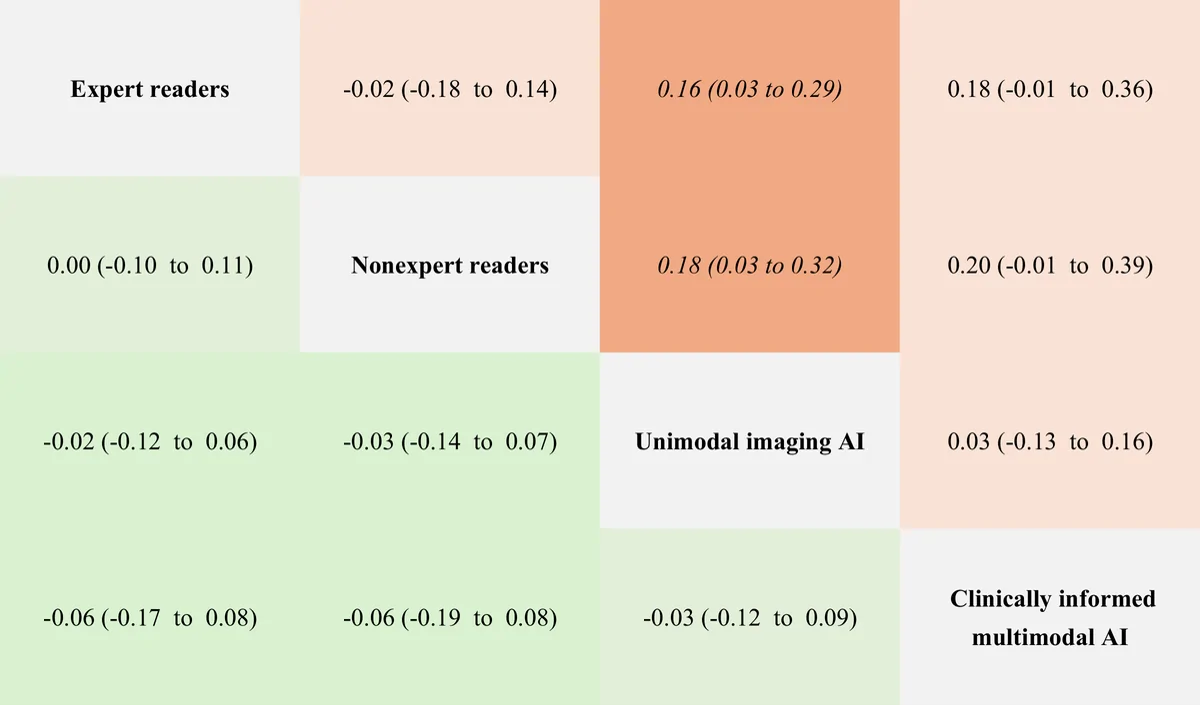
League table of absolute differences (the difference on the absolute probability scale, not the mathematical absolute value) in sensitivity and specificity between diagnostic paradigms. Values are posterior means with 95% credible intervals. Upper-right cells show sensitivity differences and lower-left cells show specificity differences, calculated as the column-defining paradigm minus the row-defining paradigm. Accordingly, a value greater than 0.00 indicates higher sensitivity (upper-right cells) or specificity (lower-left cells) for the column-defining diagnostic paradigm. Positive values indicate higher sensitivity or specificity for the column-defining paradigm. Estimates with 95% credible intervals excluding 0 are shown in italics. These comparisons should not be interpreted as product rankings or evidence of clinical superiority. AI: artificial intelligence.

SUCRA rankings were interpreted as descriptive summaries of diagnostic hierarchy rather than definitive evidence of clinical superiority. Clinically informed multimodal AI had the highest SUCRA values for sensitivity (87.8%), specificity (83.3%), and DOR (94.8%), followed by unimodal imaging AI for sensitivity (76.3%) and DOR (70.2%). Because clinically informed multimodal AI was supported by only 30% (3/10) of the studies and relied entirely on indirect evidence for comparisons with human readers, its highest point estimates should be treated as exploratory rather than as proof of clinical superiority.

### Heterogeneity, Threshold Effect, and Meta-Regression

Between-study heterogeneity (τ) on the logit scale was 0.49 (95% credible interval 0.10-0.91) for sensitivity, 0.61 (95% credible interval 0.12-1.06) for specificity, and 0.51 (95% credible interval 0.17-0.95) for log-DOR, with relatively wide credible intervals indicating limited precision. The sensitivity-specificity correlation was also imprecisely estimated (ρ=−0.58, 95% credible interval −0.97 to 0.34), precluding a firm conclusion regarding a clinically meaningful threshold-related effect. All 4 meta-regression covariates (NCCT slice thickness, M2 segment inclusion, validation strategy, and algorithm origin) had 95% credible intervals crossing 0 (Table S18 in Multimedia Appendix 1). Given the limited statistical power of 10 studies and the single laboratory-developed AI row for the commercial vs laboratory-developed AI comparison, these analyses should be considered exploratory and should not be interpreted as evidence that these factors have no influence on diagnostic performance.

### Sensitivity Analyses

Across 6 sensitivity analyses (Table S19 in Multimedia Appendix 1), the exploratory DOR hierarchy remained broadly stable. Sensitivity analyses 1 to 5 addressed validation design, anterior circulation restriction, algorithm origin, M2 inclusion, dual subset handling, and mixed or nonconcurrent reference standard characteristics. Sensitivity analysis 6, which replaced the primary uniform(0, 2) between-study SD priors with half-normal(0, 1) priors, produced no material change in node-level sensitivity, specificity, or DOR estimates or the exploratory diagnostic hierarchy. AI diagnostic nodes remained directionally favored over human reader nodes for sensitivity and DOR, whereas specificity differences remained unclear.

### Clinical Utility and Publication Bias

Clinical utility was primarily illustrated at a 20% pretest probability, approximating suspected AIS or emergency triage populations. Per 1000 patients, unimodal imaging AI yielded approximately 156 true positives, 44 false negatives, 92 false positives, and 708 true negatives compared with 125, 75, 112, and 688 for expert readers, respectively, and 121, 79, 113, and 687 for nonexpert readers, respectively. Corresponding absolute outcomes at 30% and 49.8% pretest probabilities are provided in Table S20 in Multimedia Appendix 1, with Fagan nomograms for all 3 scenarios shown in Figures S13 to S15 in Multimedia Appendix 1. The 49.8% scenario represents the enriched median prevalence of the included validation cohorts rather than routine emergency triage prevalence. The Deeks test was uninformative because of the small number of studies (*P*=.76; Figure S16 [41] in Multimedia Appendix 1).

## Discussion

### Principal Findings

In this systematic review and Bayesian DTA-NMA of 10 studies and 3632 patients, NCCT-based AI showed a comparative sensitivity signal for emergency anterior circulation LVO triage. Sensitivity and false negative reduction rather than specificity were the clearest dimensions of potential clinical value. For context, these pooled sensitivities compare favorably with the sensitivity of approximately 52% for the hyperdense artery sign [10], suggesting that AI-assisted NCCT assessment may improve LVO recognition beyond reliance on this conventional visual marker alone. The relative evidence was strongest for NCCT-only AI, whereas the higher point estimates for clinically informed multimodal AI remained exploratory because that node was sparse and relied entirely on indirect evidence for comparisons with human readers. This review extends prior syntheses of NCCT-based AI for LVO and commercial platforms by isolating the NCCT-before-CTA decision point and embedding human reader comparators within the same evidence network [13,14]. Overall, the evidence supports potential triage assistance, but diagnostic certainty should not be conflated with implementation certainty. The GRADE ratings in this review apply to diagnostic accuracy estimates in validation cohorts, not to workflow effectiveness, transfer appropriateness, EVT delivery, or patient outcomes.

### Clinical Interpretation

CTA remains the first-line noninvasive confirmatory test for anterior circulation LVO, and DSA remains the definitive invasive angiographic reference standard. NCCT-based AI should therefore not be framed as a replacement for CTA, a rule-out test, or an autonomous transfer decision system. Its clinically plausible role is earlier in the pathway: a human-in-the-loop signal that may prompt urgent image review, CTA prioritization, tele-stroke consultation, stroke team notification, or transfer discussion when vascular imaging is delayed or operationally constrained. Any reduction in missed LVOs must be balanced against false positive escalation, which may increase unnecessary CTA and contrast exposure, avoidable clinical activations or transfer discussions, workload, alert fatigue, and workflow disruption. AI-positive signals should therefore accelerate appropriate vascular confirmation rather than substitute for it.

### Workflow and Node Interpretation

The network nodes should be interpreted as workflow functions rather than product rankings. Expert readers provide a specialist benchmark, whereas nonexpert readers approximate first-contact or off-hours interpretation. These are the settings in which escalation is most likely to be delayed. Such settings may also be particularly susceptible to automation bias if AI output is given disproportionate weight during subsequent image interpretation or clinical decision-making, potentially reducing independent critical assessment of the NCCT. This concern may be especially relevant for junior or nonspecialist readers, who had the lowest pooled sensitivity in our network. However, the included retrospective validation studies did not evaluate clinician reliance on AI output, override behavior, or changes in unaided interpretation after AI exposure; therefore, the magnitude of automation bias cannot be quantified from the current evidence. NCCT-based AI should consequently remain an adjunctive human-in-the-loop prompt rather than a substitute for independent clinical assessment and confirmatory vascular imaging. From an operational perspective, unimodal imaging AI can run immediately after image acquisition and does not depend on bedside data entry. The higher point estimates observed for clinically informed multimodal AI may partly reflect the incorporation of additional clinical information, including the NIHSS in all 3 included models and symptom onset time in 1. However, the current network cannot isolate the incremental contribution of these variables from other between-study differences, and this apparent advantage remains uncertain and hypothesis generating because the node was supported by only 3 studies and relied entirely on indirect evidence for comparisons with human readers. The relevant clinical question is therefore not whether AI outperforms ideal specialist interpretation under controlled conditions but whether an early signal can accelerate vascular imaging, specialist review, or transfer discussion in time-sensitive workflows.

### Strengths and Limitations

The restrictive eligibility criteria made the network smaller but more interpretable. By requiring validation independent of model development, NCCT input, anterior circulation LVO, within-study head-to-head comparisons, and reconstructible 2 × 2 data, the review prioritized methodological comparability and interpretability over breadth. Accordingly, the included evidence represents comparative NCCT-based validation studies rather than the complete validation literature for NCCT-based LVO detection. The resulting evidence can frame a plausible escalation role, but it cannot estimate product-specific performance, workflow effectiveness, or patient benefit with precision. Generalizability and indirect comparison validity remain constrained by retrospective enriched validation cohorts and by residual variation in transitivity-related modifiers, including reader expertise, target vessel composition, NCCT acquisition parameters, reference standard characteristics, validation design, and AI input variables. Statistical network connectivity does not necessarily establish clinical exchangeability; accordingly, indirect comparisons should be interpreted as directional comparative signals rather than definitive evidence that AI intrinsically outperforms human readers. The sparse network also limited the power to detect inconsistency, particularly for comparisons involving the multimodal AI node. Shared cohort-level effects were modeled for studies evaluating AI and human readers in the same patients, but aggregate 2 × 2 data precluded explicit modeling of within-patient AI-reader correlation. In addition, category-level averaging of multiple readers may have attenuated interreader variability, and the available data did not support a consistent reader-level sensitivity analysis. The robustness of the diagnostic hierarchy to an alternative half-normal heterogeneity prior supports statistical stability of the Bayesian synthesis, but it does not overcome the need for prospective workflow validation. Together, these constraints define where prospective evaluation must occur: lower-prevalence emergency populations; CTA-constrained or off-hours pathways; sites with different scanner protocols; and predefined action pathways linking an AI-positive NCCT alert to vascular imaging, tele-stroke consultation, or transfer discussion.

### Future Directions

Future studies should move from stand-alone diagnostic accuracy to pathway effects. Deployment evaluations should predefine how an AI-positive NCCT alert is acted on and measure picture archiving and communication system integration, alert latency, clinician response, override behavior, false positive transfer burden, alert fatigue, NIHSS data entry burden, site-level generalizability, and equity of access [43]. Clinical deployment also requires appropriate IT infrastructure; integration with existing imaging and stroke workflows; continued clinician oversight; and, for multimodal systems, structured electronic health record interfaces or other standardized real-time access to relevant clinical variables. Early real-world studies have begun to evaluate NCCT-based AI implementation, whereas a cluster randomized clinical trial of a related CTA-based LVO workflow suggested that AI-assisted notification may shorten treatment times. However, prospective evidence demonstrating improved patient outcomes specifically from NCCT-based AI remains limited [44,45]. Prospective multicenter studies should also prespecify subgroup analyses by age, sex, stroke severity, scanner manufacturer, imaging protocol, hospital type, geographic setting, and relevant demographic groups to assess whether diagnostic performance and clinical utility are consistent across patient populations and implementation environments. Stroke-specific end points should include door-to-CTA time, door-in-door-out time, door-to-puncture time, transfer appropriateness, and 90-day modified Rankin Scale outcomes. Without predefined action pathways, improved diagnostic sensitivity may not translate into faster reperfusion or better functional outcomes. Future reports should also follow current AI-specific prediction model and diagnostic accuracy reporting guidelines [46,47].

### Conclusions

Taken together, the current evidence supports prospective evaluation of NCCT-based AI as a bounded escalation and prioritization signal before or alongside confirmatory vascular imaging, with the clearest comparative signal observed for unimodal imaging AI sensitivity. The current evidence does not support CTA replacement, LVO rule-out, autonomous transfer decisions, or EVT decisions. Prospective workflow-integrated validation is required before accuracy evidence can justify deployment claims, reimbursement decisions, or protocolized changes in stroke triage pathways.

## Appendix

Multimedia Appendix 1 Reporting checklists, search strategies, study characteristics, risk-of-bias and certainty assessments, and supplementary analyses for the Bayesian diagnostic test accuracy network meta-analysis.

## Abbreviations

AIS: acute ischemic stroke
CTA: computed tomography angiography
DIC: deviance information criterion
DOR: diagnostic odds ratio
DSA: digital subtraction angiography
DTA-NMA: diagnostic test accuracy network meta-analysis
EVT: endovascular thrombectomy
GRADE: Grading of Recommendations Assessment, Development, and Evaluation
LVO: large vessel occlusion
MCMC: Markov chain Monte Carlo
MRA: magnetic resonance angiography
NCCT: noncontrast computed tomography
NIHSS: National Institutes of Health Stroke Scale
PRISMA: Preferred Reporting Items for Systematic Reviews and Meta-Analyses
PRISMA-DTA: Preferred Reporting Items for Systematic Reviews and Meta-Analyses extension for diagnostic test accuracy
PRISMA-NMA: Preferred Reporting Items for Systematic Reviews and Meta-Analyses extension for network meta-analyses
PRISMA-S: Preferred Reporting Items for Systematic Reviews and Meta-Analyses literature search extension
PROBAST+AI: Prediction Model Risk of Bias Assessment Tool for Artificial Intelligence
QUADAS-3: Quality Assessment of Diagnostic Accuracy Studies–3
rDOR: relative diagnostic odds ratio
STRATIS: Systematic Evaluation of Patients Treated With Neurothrombectomy Devices for Acute Ischemic Stroke
SUCRA: surface under the cumulative ranking curve

## Data Availability

All extracted aggregate data required to reproduce this review are provided in the main text, tables, figures, Multimedia Appendix 1, and the public code repository. The R and Just Another Gibbs Sampler scripts used for Bayesian diagnostic test accuracy network meta-analysis, convergence diagnostics, and sensitivity analyses are available at GitHub [48].
