# Supplementary material for: AI Decision Support for Emergency Triage of Large Vessel Occlusion Using Noncontrast Computed Tomography: Systematic Review and Bayesian Diagnostic Test Accuracy Network Meta-Analysis
Source: J Med Internet Res. 2026 Sep 24;28:e105068. doi: 10.2196/105068 (PMC13693864; doi:10.2196/105068)
Supplement: Supplementary file 1 [file jmir_v28i1e105068_app1.docx]

**Artificial Intelligence Decision Support for Emergency Triage of Large Vessel Occlusion Using Noncontrast Computed Tomography: A Systematic Review and Bayesian Diagnostic Test Accuracy Network Meta-Analysis**

(Supplementary Materials)

**Table of Contents**

[Supplementary Table 1. Preferred Reporting Items for Systematic Reviews and Meta-Analyses of Diagnostic Test Accuracy (PRISMA-DTA) Checklist.	4](#_Toc238172608)

[Supplementary Table 2. PRISMA NMA Checklist of Items to Include When Reporting A Systematic Review Involving a Network Meta-analysis	7](#_Toc238172609)

[Supplementary Table 3. PRISMA 2020 for Abstracts Checklist	12](#_Toc238172610)

[Supplementary Table 4. PRISMA-S Checklist.	13](#_Toc238172611)

[Supplementary Table 5. Search strategy in PubMed, Embase, Web of Science, and Cochrane Library.	15](#_Toc238172612)

[Supplementary Table 6. Single-arm NCCT-based AI studies excluded at full-text assessment and reasons for exclusion.	18](#_Toc238172613)

[Supplementary Table 7. Source-reported reader expertise and derivation of human-reader 2×2 data used in the DTA-NMA.	19](#_Toc238172614)

[Supplementary Table 8. Baseline Clinical Demographics (Validations).	22](#_Toc238172615)

[Supplementary Table 9. CT Acquisition Parameters and Image Analysis Methodologies.	23](#_Toc238172616)

[Supplementary Table 10. Domain-level assessment of model development using PROBAST+AI.	24](#_Toc238172617)

[Supplementary Table 11. Domain-level assessment of model validation using PROBAST+AI.	26](#_Toc238172618)

[Supplementary Table 12. QUADAS-3 risk-of-bias and applicability assessment of included diagnostic accuracy studies.	28](#_Toc238172619)

[Supplementary Table 13. GRADE certainty assessment for pooled sensitivity and specificity estimates.	30](#_Toc238172620)

[Supplementary Table 14. GRADE certainty assessment for major comparative diagnostic accuracy estimates.	32](#_Toc238172621)

[Supplementary Table 15. League table of rDORs.	34](#_Toc238172622)

[Supplementary Table 16. Study-level potential effect modifiers relevant to the transitivity assumption across diagnostic nodes.	35](#_Toc238172623)

[Supplementary Table 17. Direct and indirect evidence structure and local inconsistency assessment.	37](#_Toc238172624)

[Supplementary Table 18. Bayesian meta-regression analysis.	38](#_Toc238172625)

[Supplementary Table 19. Sensitivity analyses.	39](#_Toc238172626)

[Supplementary Table 20. Absolute diagnostic outcomes per 1000 patients across clinically relevant LVO prevalence scenarios.	41](#_Toc238172627)

[Supplementary Figure 1. Forest plots of sensitivity, specificity, and diagnostic odds ratio for individual studies in Node 1 (Expert Readers).	42](#_Toc238172628)

[Supplementary Figure 2. Forest plots of sensitivity, specificity, and diagnostic odds ratio for individual studies in Node 2 (Non-Expert Readers).	43](#_Toc238172629)

[Supplementary Figure 3. Forest plots of sensitivity, specificity, and diagnostic odds ratio for individual studies in Node 3 (Unimodal Imaging AI).	44](#_Toc238172630)

[Supplementary Figure 4. Forest plots of sensitivity, specificity, and diagnostic odds ratio for individual studies in Node 4 (Clinically-Informed Multimodal AI).	45](#_Toc238172631)

[Supplementary Figure 5. Markov chain Monte Carlo trace plots for convergence diagnostics of the Bayesian network meta-analysis model.	46](#_Toc238172632)

[Supplementary Figure 6. Markov chain Monte Carlo posterior density overlays for convergence diagnostics of the Bayesian network meta-analysis model.	47](#_Toc238172633)

[Supplementary Figure 7. Design-by-treatment interaction heatmap for assessment of statistical inconsistency in the Bayesian network meta-analysis.	48](#_Toc238172634)

[Supplementary Figure 8. Node-splitting assessment of local inconsistency in the Bayesian diagnostic test accuracy network meta-analysis.	49](#_Toc238172635)

[Supplementary Figure 9. QUADAS-3 risk-of-bias and applicability judgments.	50](#_Toc238172636)

[Supplementary Figure 10. Posterior density distributions of pooled sensitivity and specificity from the Bayesian network meta-analysis main analysis.	51](#_Toc238172637)

[Supplementary Figure 11. Pooled sensitivity and specificity from the Bayesian network meta-analysis main analysis.	52](#_Toc238172638)

[Supplementary Figure 12. Pooled diagnostic odds ratio from the Bayesian network meta-analysis main analysis.	53](#_Toc238172639)

[Supplementary Figure 13. Node-specific Fagan nomograms at a pretest probability of 20% for suspected AIS or emergency department triage populations.	54](#_Toc238172640)

[Supplementary Figure 14. Node-specific Fagan nomograms at a pretest probability of 30% for confirmed or high-suspicion AIS populations.	55](#_Toc238172641)

[Supplementary Figure 15. Node-specific Fagan nomograms at a pretest probability of 49.8% (median LVO prevalence across included validation cohorts).	56](#_Toc238172642)

[Supplementary Figure 16. Deeks' funnel plot asymmetry test for assessment of publication bias.	57](#_Toc238172643)

## Supplementary Table 1. Preferred Reporting Items for Systematic Reviews and Meta-Analyses of Diagnostic Test Accuracy (PRISMA-DTA) Checklist.

| **TITLE/ABSTRACT** | | | **Reported on page #** |
| --- | --- | --- | --- |
| **Title** | 1 | Identify the report as a systematic review (+/−meta-analysis) of diagnostic test accuracy (DTA) studies | p. 1 |
| **Abstract** | 2 | See the PRISMA for Abstracts checklist. | pp. 2-3 |
| **INTRODUCTION** | | |  |
| **Rationale** | 3 | Describe the rationale for the review in the context of what is already known | pp. 4-5 |
| **Clinical role of index test** | D1 | State the scientific and clinical background, including the intended use and clinical role of the index test, and if applicable, the rationale for minimally acceptable test accuracy (or minimum difference in accuracy for comparative design) | pp. 4-5; Figure 1 (p. 5) |
| **Objectives** | 4 | Provide an explicit statement of question(s) being addressed in terms of participants, index test(s), and target condition(s) | p. 5 |
| **METHODS** | | |  |
| **Protocol and**  **registration** | 5 | Indicate if a review protocol exists, if and where it can be accessed (e.g., Web address), and, if available, provide registration information including registration number. | p. 6 |
| **Eligibility criteria** | 6 | Specify study characteristics (participants, setting, index test(s), reference standard(s), target condition(s), and study design) and report characteristics (e.g., years considered, language, publication status) used as criteria for eligibility, giving rationale. | pp. 6-9 |
| **Information sources** | 7 | Describe all information sources (e.g., databases with dates of coverage, contact with study authors to identify additional studies) in the search and date last searched. | p. 6 |
| **Search** | 8 | Present full search strategies for all electronic databases and other sources searched, including any limits used, such that they could be repeated. | p. 6; Supplementary Table 5 (pp. 15-17) |
| **Selection process** | 9 | State the process for selecting studies (i.e., screening, eligibility, included in systematic review, and, if applicable, included in the meta-analysis). | p. 6; Figure 2 (pp. 13-14) |
| **Data collection process** | 10 | Describe method of data extraction from reports (e.g., piloted forms, independently, in duplicate) and any processes for obtaining and confirming data from investigators. | p. 9 |
| **Definitions for data extraction** | 11 | Provide definitions used in data extraction and classifications of target condition(s), index test(s), reference standard(s) and other characteristics (e.g., study design, clinical setting). | pp. 7-9; Tables 1-2; Supplementary Tables 7–9 (pp. 19-23) |
| **Risk of bias and**  **applicability** | 12 | Describe methods used for assessing risk of bias in individual studies and concerns regarding the applicability to the review question. | pp. 9-10; Figure 4 (p. 15); Supplementary Tables 10–12 (pp. 24, 26, 28–29); Figure 9 (p. 50) |
| **Diagnostic accuracy measures** | 13 | State the principal diagnostic accuracy measure(s) reported (e.g., sensitivity, specificity) and state the unit of assessment (e.g., per-patient, per-lesion). | p. 10 |
| **Synthesis of results** | 12 | Describe methods of handling data, combining results of studies and describing variability between studies. This could include, but is not limited to: a) handling of multiple definitions of target condition. b) handling of multiple thresholds of test positivity, c) handling multiple index test readers, d) handling of indeterminate test results, e) grouping and comparing tests, f) handling of different reference standards | pp. 9-13 |
| **Meta-analysis** | D2 | Report the statistical methods used for meta-analyses, if performed. | pp. 10-13 |
| **Additional analyses** | 16 | Describe methods of additional analyses (e.g., sensitivity or subgroup analyses, meta-regression), if done, indicating which were pre-specified. | pp. 12-13; Supplementary Tables 18–19 (pp. 38-40) |
| **RESULTS** | | |  |
| **Study selection** | 17 | Provide numbers of studies screened, assessed for eligibility, included in the review (and included in meta-analysis, if applicable) with reasons for exclusions at each stage, ideally with a flow diagram. | p. 13; Figure 2 (pp. 13-14) |
| **Study characteristics** | 18 | For each included study provide citations and present key characteristics including: a) participant characteristics (presentation, prior testing), b) clinical setting, c) study design, d) target condition definition, e) index test, f) reference standard, g) sample size, h) funding sources | pp. 13-14; Table 1; Supplementary Tables 8–9 (pp. 22-23) |
| **Risk of bias in studies** | 19 | Present evaluation of risk of bias and concerns regarding applicability for each study. | pp. 15-16; Figure 4 (p. 15); Supplementary Tables 10–12 (pp. 24, 26, 28–29); Figure 9 (p. 50) |
| **Results of individual studies** | 20 | For each analysis in each study (e.g., unique combination of index test, reference standard, and positivity threshold) report 2×2 data (TP, FP, FN, TN) with estimates of diagnostic accuracy and confidence intervals, ideally with a forest or receiver operator characteristic (ROC) plot. | Tables 1-2; Supplementary Figures 1–4 (pp. 42-45) |
| **Synthesis of results** | 22 | Describe test accuracy, including variability; if meta-analysis was done, include results and confidence intervals. | pp. 16-17; Figures 5-6 (p. 16); Supplementary Tables 13 and 15 (pp. 30-31, 34); Supplementary Figures 10–12 (pp. 51-53) |
| **Additional analysis** | 23 | Give results of additional analyses, if done (e.g., sensitivity or subgroup analyses, meta-regression; analysis of index test: failure rates, proportion of inconclusive results, adverse events). | pp. 17-19; Supplementary Tables 18–19 (pp. 38-40); Supplementary Figures 13–16 (pp. 54-57) |
| **DISCUSSION** | | |  |
| **Summary of evidence** | 24 | Summarize the main findings including the strength of evidence. | pp. 19-21 |
| **Limitations** | 25 | Discuss limitations from included studies (e.g., risk of bias and concerns regarding applicability) and from the review process (e.g., incomplete retrieval of identified research). | pp. 21-23 |
| **Conclusions** | 26 | Provide a general interpretation of the results in the context of other evidence. Discuss implications for future research and clinical practice (e.g., the intended use and clinical role of the index test). | p. 24 |
| **FUNDING** | | |  |
| **Funding** | 26 | For the systematic review, describe the sources of funding and other support and the role of the funders. | pp. 24-25 |

Salameh, Jean-Paul et al. “Preferred reporting items for systematic review and meta-analysis of diagnostic test accuracy studies (PRISMA-DTA): explanation, elaboration, and checklist.” *BMJ (Clinical research ed.)* vol. 370 m2632. 14 Aug. 2020, doi:10.1136/bmj.m2632

## Supplementary Table 2. PRISMA NMA Checklist of Items to Include When Reporting A Systematic Review Involving a Network Meta-analysis

| **Section/Topic** | **Item #** | **Checklist Item** | **Reported on Page #** |
| --- | --- | --- | --- |
| **TITLE** | | |  |
| **Title** | 1 | Identify the report as a systematic review *incorporating a network meta-analysis (or related form of meta-analysis).* | p. 1 |
| **ABSTRACT** | | |  |
| **Structured summary** | 2 | Provide a structured summary including, as applicable:  **Background:** main objectives  **Methods:** data sources; study eligibility criteria, participants, and interventions; study appraisal; and *synthesis methods, such as network meta-analysis.*  **Results:** number of studies and participants identified; summary estimates with corresponding confidence/credible intervals; *treatment rankings may also be discussed. Authors may choose to summarize pairwise comparisons against a chosen treatment included in their analyses for brevity.*  **Discussion/Conclusions:** limitations; conclusions and implications of findings.  **Other:** primary source of funding; systematic review registration number with registry name. | pp. 2-3 |
| **INTRODUCTION** | | |  |
| **Rationale** | 3 | Describe the rationale for the review in the context of what is already known*, including mention of why a network meta-analysis has been conducted.* | pp. 4-5 |
| **Objectives** | 4 | Provide an explicit statement of questions being addressed, with reference to participants, interventions, comparisons, outcomes, and study design (PICOS). | p. 5 |
| **METHODS** | | |  |
| **Protocol and registration** | 5 | Indicate whether a review protocol exists and if and where it can be accessed (e.g., Web address); and, if available, provide registration information, including registration number. | p. 6 |
| **Eligibility criteria** | 6 | Specify study characteristics (e.g., PICOS, length of follow-up) and report characteristics (e.g., years considered, language, publication status) used as criteria for eligibility, giving rationale. *Clearly describe eligible treatments included in the treatment network, and note whether any have been clustered or merged into the same node (with justification).* | pp. 6-9 |
| **Information sources** | 7 | Describe all information sources (e.g., databases with dates of coverage, contact with study authors to identify additional studies) in the search and date last searched. | p. 6 |
| **Search** | 8 | Present full electronic search strategy for at least one database, including any limits used, such that it could be repeated. | p. 6; Supplementary Table 5 (pp. 15-17) |
| **Study selection** | 9 | State the process for selecting studies (i.e., screening, eligibility, included in systematic review, and, if applicable, included in the meta-analysis). | p. 6; Figure 2 (pp. 13-14) |
| **Data collection process** | 10 | Describe method of data extraction from reports (e.g., piloted forms, independently, in duplicate) and any processes for obtaining and confirming data from investigators. | p. 9 |
| **Data items** | 11 | List and define all variables for which data were sought (e.g., PICOS, funding sources) and any assumptions and simplifications made. | pp. 7-9; Tables 1-2; Supplementary Tables 7–9 (pp. 19-23) |
| **Geometry of the network** | **S1** | Describe methods used to explore the geometry of the treatment network under study and potential biases related to it. This should include how the evidence base has been graphically summarized for presentation, and what characteristics were compiled and used to describe the evidence base to readers. | pp. 10-13 and p. 14; Figure 3 (p. 14) |
| **Risk of bias within individual studies** | 12 | Describe methods used for assessing risk of bias of individual studies (including specification of whether this was done at the study or outcome level), and how this information is to be used in any data synthesis. | pp. 9-10; Supplementary Tables 10–12 (pp. 24, 26, 28–29); Figure 9 (p. 50) |
| **Summary measures** | 13 | State the principal summary measures (e.g., risk ratio, difference in means). *Also describe the use of additional summary measures assessed, such as treatment rankings and surface under the cumulative ranking curve (SUCRA) values, as well as modified approaches used to present summary findings from meta-analyses.* | p. 10 |
| **Planned methods of analysis** | 14 | Describe the methods of handling data and combining results of studies for each network meta-analysis. This should include, but not be limited to:   - *Handling of multi-arm trials;* - *Selection of variance structure;* - *Selection of prior distributions in Bayesian analyses; and* - *Assessment of model fit.* | pp. 10-13 |
| **Assessment of Inconsistency** | **S2** | Describe the statistical methods used to evaluate the agreement of direct and indirect evidence in the treatment network(s) studied. Describe efforts taken to address its presence when found. | pp. 11-13 and p. 15; Supplementary Figures 7–8 (pp. 48-49) |
| **Risk of bias across studies** | 15 | Specify any assessment of risk of bias that may affect the cumulative evidence (e.g., publication bias, selective reporting within studies). | pp. 12 and 19; Supplementary Figure 16 (p. 57) |
| **Additional analyses** | 16 | Describe methods of additional analyses if done, indicating which were pre-specified. This may include, but not be limited to, the following:   - Sensitivity or subgroup analyses; - Meta-regression analyses; - *Alternative formulations of the treatment network; and* - *Use of alternative prior distributions for Bayesian analyses (if applicable).* | pp. 12-13 |
| **RESULTS†** | | |  |
| **Study selection** | 17 | Give numbers of studies screened, assessed for eligibility, and included in the review, with reasons for exclusions at each stage, ideally with a flow diagram. | p. 13; Figure 2 (pp. 13-14) |
| **Presentation of network structure** | **S3** | Provide a network graph of the included studies to enable visualization of the geometry of the treatment network. | pp. 14-15; Figure 3 (p. 14) |
| **Summary of network geometry** | **S4** | Provide a brief overview of characteristics of the treatment network. This may include commentary on the abundance of trials and randomized patients for the different interventions and pairwise comparisons in the network, gaps of evidence in the treatment network, and potential biases reflected by the network structure. | pp. 14-15; Figure 3 (p. 14) |
| **Study characteristics** | 18 | For each study, present characteristics for which data were extracted (e.g., study size, PICOS, follow-up period) and provide the citations. | pp. 13-14; Table 1; Supplementary Tables 8–9 (pp. 22-23) |
| **Risk of bias within studies** | 19 | Present data on risk of bias of each study and, if available, any outcome level assessment. | pp. 15-16; Figure 4 (p. 15); Supplementary Tables 10–12 (pp. 24, 26, 28–29); Figure 9 (p. 50) |
| **Results of individual studies** | 20 | For all outcomes considered (benefits or harms), present, for each study: 1) simple summary data for each intervention group, and 2) effect estimates and confidence intervals. *Modified approaches may be needed to deal with information from larger networks.* | Tables 1-2; Supplementary Figures 1–4 (pp. 42-45) |
| **Synthesis of results** | 21 | Present results of each meta-analysis done, including confidence/credible intervals. *In larger networks, authors may focus on comparisons versus a particular comparator (e.g. placebo or standard care), with full findings presented in an appendix. League tables and forest plots may be considered to summarize pairwise comparisons.* If additional summary measures were explored (such as treatment rankings), these should also be presented. | pp. 16-17; Figures 5-6 (p. 16); Supplementary Tables 13 and 15 (pp. 30-31, 34); Supplementary Figures 10–12 (pp. 51-53) |
| **Exploration for inconsistency** | **S5** | Describe results from investigations of inconsistency. This may include such information as measures of model fit to compare consistency and inconsistency models, *P* values from statistical tests, or summary of inconsistency estimates from different parts of the treatment network. | p. 15; Supplementary Figures 7–8 (pp. 48-49) |
| **Risk of bias across studies** | 22 | Present results of any assessment of risk of bias across studies for the evidence base being studied. | p. 19; Supplementary Figure 16 (p. 57) |
| **Results of additional analyses** | 23 | Give results of additional analyses, if done (e.g., sensitivity or subgroup analyses, meta-regression analyses*, alternative network geometries studied, alternative choice of prior distributions for Bayesian analyses,* and so forth). | pp. 17-19; Supplementary Tables 18–19 (pp. 38-40); Supplementary Figures 13–16 (pp. 54-57) |
| **DISCUSSION** | | |  |
| **Summary of evidence** | 24 | Summarize the main findings, including the strength of evidence for each main outcome; consider their relevance to key groups (e.g., healthcare providers, users, and policy-makers). | pp. 19-21 |
| **Limitations** | 25 | Discuss limitations at study and outcome level (e.g., risk of bias), and at review level (e.g., incomplete retrieval of identified research, reporting bias). *Comment on the validity of the assumptions, such as transitivity and consistency. Comment on any concerns regarding network geometry (e.g., avoidance of certain comparisons).* | pp. 21-23 |
| **Conclusions** | 26 | Provide a general interpretation of the results in the context of other evidence, and implications for future research. | p. 24 |
| **FUNDING** | | |  |
| **Funding** | 27 | Describe sources of funding for the systematic review and other support (e.g., supply of data); role of funders for the systematic review. This should also include information regarding whether funding has been received from manufacturers of treatments in the network and/or whether some of the authors are content experts with professional conflicts of interest that could affect use of treatments in the network. | pp. 24-25 |

**Abbreviation:** PICOS: population, intervention, comparators, outcomes, study design.

* Text in italics indicates wording specific to reporting of network meta-analyses that has been added to guidance from the PRISMA statement.

† Authors may wish to plan for use of appendices to present all relevant information in full detail for items in this section.

## Supplementary Table 3. PRISMA 2020 for Abstracts Checklist

| **Section and Topic** | **Item #** | **Checklist item** | Reported on page # |
| --- | --- | --- | --- |
|  | | |  |
| TITLE | | |  |
| Title | 1 | Identify the report as a systematic review. | p. 1 |
| BACKGROUND | | |  |
| Objectives | 2 | Provide an explicit statement of the main objective(s) or question(s) the review addresses. | p. 2 |
| METHODS | | |  |
| Eligibility criteria | 3 | Specify the inclusion and exclusion criteria for the review. | p. 2 |
| Information sources | 4 | Specify the information sources (e.g. databases, registers) used to identify studies and the date when each was last searched. | p. 2 |
| Risk of bias | 5 | Specify the methods used to assess risk of bias in the included studies. | p. 2 |
| Synthesis of results | 6 | Specify the methods used to present and synthesise results. | p. 2 |
| RESULTS | | |  |
| Included studies | 7 | Give the total number of included studies and participants and summarize relevant characteristics of studies. | pp. 2-3 |
| Synthesis of results | 8 | Present results for main outcomes, preferably indicating the number of included studies and participants for each. If meta-analysis was done, report the summary estimate and confidence/credible interval. If comparing groups, indicate the direction of the effect (i.e. which group is favored). | pp. 2-3 |
| DISCUSSION | | |  |
| Limitations of evidence | 9 | Provide a brief summary of the limitations of the evidence included in the review (e.g. study risk of bias, inconsistency and imprecision). | p. 3 |
| Interpretation | 10 | Provide a general interpretation of the results and important implications. | p. 3 |
| OTHER | | |  |
| Funding | 11 | Specify the primary source of funding for the review. | pp. 24-25 |
| Registration | 12 | Provide the register name and registration number. | p. 3 |

## Supplementary Table 4. PRISMA-S Checklist.

| Section/topic c | # | Checklist item | Location(s) Reported |
| --- | --- | --- | --- |
| INFORMATION SOURCES AND METHODS | | | |
| Database name | 1 | Name each individual database searched, stating the platform for each. | p. 6; Supplementary Table 5 (pp. 15-17) |
| Multi-database searching | 2 | If databases were searched simultaneously on a single platform, state the name of the platform, listing all of the databases searched. | Not applicable; databases searched separately |
| Study registries | 3 | List any study registries searched. | Not searched |
| Online resources and browsing | 4 | Describe any online or print source purposefully searched or browsed (e.g., tables of contents, print conference proceedings, web sites), and how this was done. | p. 6 |
| Citation searching | 5 | Indicate whether cited references or citing references were examined, and describe any methods used for locating cited/citing references (e.g., browsing reference lists, using a citation index, setting up email alerts for references citing included studies). | p. 6 |
| Contacts | 6 | Indicate whether additional studies or data were sought by contacting authors, experts, manufacturers, or others. | Not performed |
| Other methods | 7 | Describe any additional information sources or search methods used. | p. 6 |
| SEARCH STRATEGIES | | | |
| Full search strategies | 8 | Include the search strategies for each database and information source, copied and pasted exactly as run. | Supplementary Table 5 (pp. 15-17) |
| Limits and restrictions | 9 | Specify that no limits were used, or describe any limits or restrictions applied to a search (e.g., date or time period, language, study design) and provide justification for their use. | p. 6; Supplementary Table 5 (pp. 15-17) |
| Search filters | 10 | Indicate whether published search filters were used (as originally designed or modified), and if so, cite the filter(s) used. | Not used |
| Prior work | 11 | Indicate when search strategies from other literature reviews were adapted or reused for a substantive part or all of the search, citing the previous review(s). | Not adapted from prior reviews |
| Updates | 12 | Report the methods used to update the search(es) (e.g., rerunning searches, email alerts). | p. 6; Supplementary Table 5 (pp. 15-17) |
| Dates of searches | 13 | For each search strategy, provide the date when the last search occurred. | p. 6; Supplementary Table 5 (pp. 15-17) |
| PEER REVIEW | | | |
| Peer review | 14 | Describe any search peer review process. | Not performed |
| MANAGING RECORDS | | | |
| Total Records | 15 | Document the total number of records identified from each database and other information sources. | p. 13; Figure 2 (pp. 13-14) |
| Deduplication | 16 | Describe the processes and any software used to deduplicate records from multiple database searches and other information sources. | p. 13; Figure 2 (pp. 13-14) |

PRISMA-S: An Extension to the PRISMA Statement for Reporting Literature Searches in Systematic Reviews

Rethlefsen ML, Kirtley S, Waffenschmidt S, Ayala AP, Moher D, Page MJ, Koffel JB, PRISMA-S Group.

Last updated February 27, 2020.

## Supplementary Table 5. Search strategy in PubMed, Embase, Web of Science, and Cochrane Library.

| **Database** | **Search strategy** | **Filters and Limits** | **Number of records** |
| --- | --- | --- | --- |
| PubMed | ("Ischemic Stroke"[Mesh] OR "Brain Ischemia"[Mesh] OR "Stroke"[Mesh] OR "ischemic stroke*"[Title/Abstract] OR AIS[Title/Abstract] OR "acute stroke*"[Title/Abstract] OR "brain infarction"[Title/Abstract] OR "cerebral infarction"[Title/Abstract])  AND  ("large vessel occlusion*"[Title/Abstract] OR "major vessel occlusion*"[Title/Abstract] OR LVO[Title/Abstract] OR ELVO[Title/Abstract] OR "proximal arterial occlusion"[Title/Abstract] OR "intracranial large artery occlusion"[Title/Abstract] OR "vessel occlusion"[Title/Abstract] OR "middle cerebral artery occlusion"[Title/Abstract] OR "MCA occlusion"[Title/Abstract] OR "carotid artery occlusion"[Title/Abstract] OR "ICA occlusion"[Title/Abstract] OR "anterior circulation occlusion"[Title/Abstract])  AND  ("Tomography, X-Ray Computed"[Mesh] OR "non-contrast CT"[Title/Abstract] OR "noncontrast CT"[Title/Abstract] OR "unenhanced CT"[Title/Abstract] OR "plain CT"[Title/Abstract] OR NCCT[Title/Abstract] OR NECT[Title/Abstract] OR "computed tomography"[Title/Abstract] OR "CT"[Title/Abstract])  AND  ("Artificial Intelligence"[Mesh] OR "Machine Learning"[Mesh] OR "Deep Learning"[Mesh] OR "Radiomics"[Mesh] OR "artificial intelligence"[Title/Abstract] OR AI[Title/Abstract] OR "machine learning"[Title/Abstract] OR "deep learning"[Title/Abstract] OR radiomic*[Title/Abstract] OR "texture analysis"[Title/Abstract] OR "neural network*"[Title/Abstract] OR CNN[Title/Abstract] OR transformer*[Title/Abstract] OR ViT[Title/Abstract] OR Swin[Title/Abstract] OR Mamba[Title/Abstract] OR "foundation model*"[Title/Abstract] OR GAN[Title/Abstract] OR "generative adversarial"[Title/Abstract] OR "support vector machine*"[Title/Abstract] OR SVM[Title/Abstract] OR "random forest*"[Title/Abstract] OR XGBoost[Title/Abstract] OR LightGBM[Title/Abstract] OR "gradient boosting"[Title/Abstract] OR "decision tree*"[Title/Abstract] OR ResNet[Title/Abstract] OR U-Net[Title/Abstract] OR DenseNet[Title/Abstract] OR VGG[Title/Abstract] OR algorithm*[Title/Abstract] OR nomogram*[Title/Abstract] OR "predictive model*"[Title/Abstract] OR "prediction model*"[Title/Abstract] OR automated[Title/Abstract] OR automatic[Title/Abstract] OR "computer-aided"[Title/Abstract] OR "computer vision"[Title/Abstract]) | No date, language, or study-type restrictions were applied; last search May 24, 2026. | 393 |
| Embase | ('ischemic stroke'/exp OR 'brain ischemia'/exp OR 'cerebrovascular accident'/exp OR 'ischemic stroke*':ab,ti OR AIS:ab,ti OR 'acute stroke*':ab,ti OR 'brain infarction':ab,ti OR 'cerebral infarction':ab,ti)  AND  ('large vessel occlusion*':ab,ti OR 'major vessel occlusion*':ab,ti OR LVO:ab,ti OR ELVO:ab,ti OR 'proximal arterial occlusion':ab,ti OR 'intracranial large artery occlusion':ab,ti OR 'vessel occlusion':ab,ti OR 'middle cerebral artery occlusion':ab,ti OR 'MCA occlusion':ab,ti OR 'carotid artery occlusion':ab,ti OR 'ICA occlusion':ab,ti OR 'anterior circulation occlusion':ab,ti)  AND  ('computer assisted tomography'/exp OR 'non-contrast CT':ab,ti OR 'noncontrast CT':ab,ti OR 'unenhanced CT':ab,ti OR 'plain CT':ab,ti OR NCCT:ab,ti OR NECT:ab,ti OR 'computed tomography':ab,ti OR 'CT':ab,ti)  AND  ('artificial intelligence'/exp OR 'machine learning'/exp OR 'deep learning'/exp OR 'radiomics'/exp OR 'artificial intelligence':ab,ti OR AI:ab,ti OR 'machine learning':ab,ti OR 'deep learning':ab,ti OR radiomic*:ab,ti OR 'texture analysis':ab,ti OR 'neural network*':ab,ti OR CNN:ab,ti OR transformer*:ab,ti OR ViT:ab,ti OR Swin:ab,ti OR Mamba:ab,ti OR 'foundation model*':ab,ti OR GAN:ab,ti OR 'generative adversarial':ab,ti OR 'support vector machine*':ab,ti OR SVM:ab,ti OR 'random forest*':ab,ti OR XGBoost:ab,ti OR LightGBM:ab,ti OR 'gradient boosting':ab,ti OR 'decision tree*':ab,ti OR ResNet:ab,ti OR U-Net:ab,ti OR DenseNet:ab,ti OR VGG:ab,ti OR algorithm*:ab,ti OR nomogram*:ab,ti OR 'predictive model*':ab,ti OR 'prediction model*':ab,ti OR automated:ab,ti OR automatic:ab,ti OR 'computer-aided':ab,ti OR 'computer vision':ab,ti) | No date, language, or study-type restrictions were applied; last search May 24, 2026. | 860 |
| Web of Science | TS=("Ischemic Stroke" OR "Brain Ischemia" OR "Stroke" OR "ischemic stroke*" OR AIS OR "acute stroke*" OR "brain infarction" OR "cerebral infarction")  AND  TS=("large vessel occlusion*" OR "major vessel occlusion*" OR LVO OR ELVO OR "proximal arterial occlusion" OR "intracranial large artery occlusion" OR "vessel occlusion" OR "middle cerebral artery occlusion" OR "MCA occlusion" OR "carotid artery occlusion" OR "ICA occlusion" OR "anterior circulation occlusion")  AND  TS=("Tomography, X-Ray Computed" OR "non-contrast CT" OR "noncontrast CT" OR "unenhanced CT" OR "plain CT" OR NCCT OR NECT OR "computed tomography" OR CT)  AND  TS=("Artificial Intelligence" OR "Machine Learning" OR "Deep Learning" OR Radiomics OR AI OR radiomic* OR "texture analysis" OR "neural network*" OR CNN OR transformer* OR ViT OR Swin OR Mamba OR "foundation model*" OR GAN OR "generative adversarial" OR "support vector machine*" OR SVM OR "random forest*" OR XGBoost OR LightGBM OR "gradient boosting" OR "decision tree*" OR ResNet OR "U-Net" OR DenseNet OR VGG OR algorithm* OR nomogram* OR "predictive model*" OR "prediction model*" OR automated OR automatic OR "computer-aided" OR "computer vision") | No date, language, or study-type restrictions were applied; last search May 24, 2026. | 486 |
| Cochrane Library | ([mh "Ischemic Stroke"] OR [mh "Brain Ischemia"] OR [mh "Stroke"] OR (ischemic NEXT stroke*):ti,ab,kw OR (ischemic NEXT stroke*):ti,ab,kw OR AIS:ti,ab,kw OR (acute NEXT stroke*):ti,ab,kw OR "brain infarction":ti,ab,kw OR "cerebral infarction":ti,ab,kw) AND ((large NEXT vessel NEXT occlusion*):ti,ab,kw OR (major NEXT vessel NEXT occlusion*):ti,ab,kw OR LVO:ti,ab,kw OR ELVO:ti,ab,kw OR "proximal arterial occlusion":ti,ab,kw OR "intracranial large artery occlusion":ti,ab,kw OR "vessel occlusion":ti,ab,kw OR "middle cerebral artery occlusion":ti,ab,kw OR "MCA occlusion":ti,ab,kw OR "carotid artery occlusion":ti,ab,kw OR "ICA occlusion":ti,ab,kw OR "anterior circulation occlusion":ti,ab,kw) AND ([mh "Tomography, X-Ray Computed"] OR "non-contrast CT":ti,ab,kw OR "noncontrast CT":ti,ab,kw OR "unenhanced CT":ti,ab,kw OR "plain CT":ti,ab,kw OR NCCT:ti,ab,kw OR NECT:ti,ab,kw OR "computed tomography":ti,ab,kw OR CT:ti,ab,kw) AND ([mh "Artificial Intelligence"] OR [mh "Machine Learning"] OR [mh "Deep Learning"] OR [mh "Radiomics"] OR "artificial intelligence":ti,ab,kw OR AI:ti,ab,kw OR "machine learning":ti,ab,kw OR "deep learning":ti,ab,kw OR radiomic*:ti,ab,kw OR "texture analysis":ti,ab,kw OR (neural NEXT network*):ti,ab,kw OR CNN:ti,ab,kw OR transformer*:ti,ab,kw OR ViT:ti,ab,kw OR Swin:ti,ab,kw OR Mamba:ti,ab,kw OR (foundation NEXT model*):ti,ab,kw OR GAN:ti,ab,kw OR "generative adversarial":ti,ab,kw OR (support NEXT vector NEXT machine*):ti,ab,kw OR SVM:ti,ab,kw OR (random NEXT forest*):ti,ab,kw OR XGBoost:ti,ab,kw OR LightGBM:ti,ab,kw OR "gradient boosting":ti,ab,kw OR (decision NEXT tree*):ti,ab,kw OR ResNet:ti,ab,kw OR "U-Net":ti,ab,kw OR DenseNet:ti,ab,kw OR VGG:ti,ab,kw OR algorithm*:ti,ab,kw OR nomogram*:ti,ab,kw OR (predictive NEXT model*):ti,ab,kw OR (prediction NEXT model*):ti,ab,kw OR automated:ti,ab,kw OR automatic:ti,ab,kw OR "computer-aided":ti,ab,kw OR "computer vision":ti,ab,kw) | No date, language, or study-type restrictions were applied; last search May 24, 2026. | 36 |

## Supplementary Table 6. Single-arm NCCT-based AI studies excluded at full-text assessment and reasons for exclusion.

| **Study** | **DOI** | **Index test** | **Validation design** | **Target condition** | **Reference standard** | **Reported validation performance** | **Exclusion rationale** |
| --- | --- | --- | --- | --- | --- | --- | --- |
| Sanders et al., 2025 | 10.1177/15910199251362073 | Methinks LVO; NCCT-only AI | Independent external validation in a U.S. multi-facility stroke network; n = 608 | ICA, M1, and M2 LVO | CTA with board-certified neuroradiologist review | Sensitivity 75%; specificity 83% | No second eligible diagnostic node for the prespecified network |
| Fussell et al., 2026 | 10.3174/ajnr.A8954 | 3D deep-learning model using high-resolution NCCT (≤ 1.0-mm source data) | Temporally independent internal test cohort; n = 1011 | Acute MCA M1/M2 occlusion | Same-day CTA | M1/M2 analysis: sensitivity 77.4%; specificity 93.6% | No second eligible diagnostic node for the prespecified network |
| You et al., 2020 | 10.3389/fninf.2020.00013 | Hierarchical multimodal ML/AI model incorporating demographic, clinical, and NCCT-derived features | Random split of the same source population: 200 training and 100 testing | Anterior-circulation LVO | Clinical/imaging adjudication by cerebrovascular specialists; CTA when available | Level-3 XGBoost: sensitivity 95.3%; specificity 68.4% | No second eligible diagnostic node; additionally, validation used a non-independent random train/test split |

**Note:** “Single-arm” denotes evaluation of fewer than two prespecified diagnostic nodes eligible for the network; comparisons among algorithms within the same prespecified node were not considered head-to-head network comparisons. Sanders et al. and Fussell et al. otherwise met the remaining eligibility criteria and were excluded solely because no second eligible diagnostic node was available. You et al. additionally used a non-independent random train/test split within the same source population and therefore would not have met the primary validation requirement even if a second eligible comparator had been available.

**Abbreviations:** AI, artificial intelligence; CTA, computed tomography angiography; DOI, digital object identifier; ICA, internal carotid artery; LVO, large vessel occlusion; M1, first segment of the middle cerebral artery; M2, second segment of the middle cerebral artery; ML, machine learning; NCCT, noncontrast computed tomography.

## Supplementary Table 7. Source-reported reader expertise and derivation of human-reader 2×2 data used in the DTA-NMA.

| **Study** | **Human-reader node classification** | **Source-reported comparator structure** | **Source-reported expertise** | **Derivation of human-reader 2×2 data used in the DTA-NMA** |
| --- | --- | --- | --- | --- |
| Lee et al. 2025 | Node 1, Expert Readers; Node 2, Non-Expert Readers | The source described 2 stroke experts, 1 board-certified neurosurgeon, 1 board-certified emergency medicine specialist, and 2 neurology residents within the observer-performance and decision framework. A senior neurosurgeon adjudicated majority-vote decisions when required. | Stroke experts had more than 10 years of clinical experience. Reader-panel experience was not fully specified. Training levels included board-certified specialists and resident-level readers. | Expert Readers: source-reported unassisted consensus-level 2×2 counts were used directly; no averaging was performed. Non-Expert Readers: reader-level TP, FP, FN, and TN counts for the three readers assigned to this category were averaged directly and rounded to whole numbers (TP=75, FP=118, FN=37, TN=247). |
| Rai et al. 2025, Subset A reader comparison | Node 1, Expert Readers; Node 2, Non-Expert Readers | Ten radiologists participated in the NCCT reader comparison: 6 general radiologists were explicitly described as nonexperts, and 4 fellowship-trained neuroradiologists were explicitly described as experts. | Expert readers were US board-certified neuroradiologists with at least 4 years of board-certified work experience. Experience years were not reported for the general radiologist group. | Source-reported iMRMC group-level sensitivity and specificity for the expert and nonexpert reader groups were used. These estimates were converted to integer 2×2 counts using 113 LVO-positive and 114 LVO-negative cases (Expert: TP=55, FP=6, FN=58, TN=108; Non-Expert: TP=53, FP=17, FN=60, TN=97). |
| Yedavalli et al. 2023 | Node 1, Expert Readers; Node 2, Non-Expert Readers | The human comparison comprised 8 general radiologists and 3 neuroradiologists. The source reported these reader groups separately and compared software performance with each group. | All 11 readers were board-certified general radiologists or neuroradiologists. Reader-group experience was not specified. | Hybrid derivation. Source-reported group-average sensitivity was used directly for each reader category, whereas category-level specificity was derived by averaging the corresponding individual-reader specificity estimates. The resulting group-level estimates were converted to integer 2×2 counts using 115 LVO-positive and 129 LVO-negative cases (Expert: TP=59, FP=17, FN=56, TN=112; Non-Expert: TP=47, FP=4, FN=68, TN=125). |
| Weyland et al. 2022 | Node 2, Non-Expert Readers | The human NCCT comparison was performed by 2 blinded neuroradiology residents. The study also evaluated Brainomix hyperdense artery sign detection software. | Human readers were neuroradiology residents with 2 and 3 years of experience. The CTA reference standard was reviewed by a board-certified neuroradiologist with 18 years of experience. | Investigator-derived reader average. Reader-level sensitivity and specificity were averaged across the two neuroradiology residents and then converted to an integer 2×2 table using 84 LVO-positive and 70 LVO-negative cases (TP=73, FP=11, FN=11, TN=59). |
| Chung et al. 2025 | Node 1, Expert Readers | The NCCT human comparison used 2 radiologists who independently reviewed a randomly selected NCCT subset for LVO while blinded to clinical information. | Both NCCT comparison readers were radiologists with more than 10 years of NCCT interpretation experience. | Investigator-derived reader average. Reader-level sensitivity and specificity were averaged across the two experienced radiologists and then converted to an integer 2×2 table using 35 LVO-positive and 30 LVO-negative cases (TP=19, FP=4, FN=16, TN=26). |
| Urra et al. 2025 | Node 1, Expert Readers; Node 2, Non-Expert Readers | The NCCT reader comparison used 2 certified neuroradiologists and 2 general radiologists. The source explicitly compared expert neuroradiologists and nonexpert general radiologists. | Certification was reported for neuroradiologists. Specific years of experience were not reported for either reader group. | Source-reported average reader-group sensitivity and specificity were used directly and converted to integer 2×2 counts using 71 LVO-positive and 118 LVO-negative cases (Expert: TP=30, FP=8, FN=41, TN=110; Non-Expert: TP=21, FP=13, FN=50, TN=105). |
| Sunwoo et al. 2026, US cohort | Node 1, Expert Readers; Node 2, Non-Expert Readers | The US external-validation cohort included 240 subjects and compared standalone AI with 5 US board-certified radiologists: 3 neuroradiologists and 2 general radiologists. The reference standard was based on vascular imaging (CTA, MRA, or DSA) within 24 hours of baseline NCCT, with mixed modalities as reported by the source publication. | The radiologists were US board certified. Reader subgroup-specific experience was not specified. | Source-reported pooled subgroup sensitivity and specificity for neuroradiologists and general radiologists were used directly and converted to integer 2×2 counts using 120 LVO-positive and 120 LVO-negative cases (Expert: TP=63, FP=16, FN=57, TN=104; Non-Expert: TP=76, FP=24, FN=44, TN=96). |
| Olive-Gadea et al. 2020 | No human-reader node assigned | The study compared automated NCCT reading with neuroradiologist visual assessment of CTA. No human NCCT reader comparator was available for node assignment. | The neuroradiologists involved in reference-standard assessment were described as experienced in stroke evaluation. Years of experience were not reported. | Not applicable; no eligible human NCCT reader arm contributed to the network. |
| Kim et al. 2024 | No human-reader node assigned | LVO status was diagnosed by an experienced neurologist and compared with independently verified stroke registry data from attending vascular neurologists. No human NCCT reader comparator was available for node assignment. | The neurologists were described as experienced or attending vascular neurologists. Years of experience were not reported. NIHSS scores were evaluated by certified physicians. | Not applicable; no eligible human NCCT reader arm contributed to the network. |
| Tolhuisen et al. 2020 | Node 1, Expert Readers; Node 2, Non-Expert Readers | The test-set ground truth was established by joint reading of 2 experts. The comparison readers were an expert neuroradiologist and a neuroradiologist in training. | Ground-truth experts had more than 5 years of experience. The expert reader was an expert neuroradiologist with more than 15 years of experience. The non-expert reader was a neuroradiologist in training with more than 4 years of experience. | No multi-reader averaging was required. Each reader category was represented by one individual reader; reader-specific diagnostic performance was converted to integer 2×2 counts using 58 LVO-positive and 45 LVO-negative cases (Expert: TP=54, FP=18, FN=4, TN=27; Non-Expert: TP=45, FP=5, FN=13, TN=40). |

**Note:** Source-reported consensus, group-average, pooled, or multireader-multicase estimates were treated as directly reported group-level results rather than investigator-derived reader averages. When no category-level estimate was reported, individual-reader results were averaged within the prespecified expertise category as detailed above. For reconstruction from sensitivity and specificity, TP and TN were calculated using the corresponding numbers of LVO-positive and LVO-negative cases and rounded to whole numbers, with FN and FP derived from the respective denominators. No noninteger TP, FP, FN, or TN counts were entered into the Bayesian binomial model. Expert Readers included senior neuroradiologists, stroke specialists, or experienced radiologists explicitly identified as expert or senior readers, or reported as having substantial NCCT interpretation experience. Non-Expert Readers included non-neuroradiology or non-stroke-specialist readers, including general radiologists, emergency physicians, junior radiology or neurology residents, or trainees. Studies without an eligible human NCCT reader comparator did not contribute Expert Reader or Non-Expert Reader nodes.

**Abbreviations:** AI, artificial intelligence; CTA, computed tomography angiography; DSA, digital subtraction angiography; DTA-NMA, diagnostic test accuracy network meta-analysis; FN, false negative; FP, false positive; LVO, large vessel occlusion; MRA, magnetic resonance angiography; NCCT, noncontrast computed tomography; NIHSS, National Institutes of Health Stroke Scale; TN, true negative; TP, true positive.

## Supplementary Table 8. Baseline Clinical Demographics (Validations).

| **Study ID** | **Cohort Type** | **Cohort N** | **LVO Prevalence (%)** | **Age (Years)** | **Male (%)** | **Baseline NIHSS** | **Onset-to-CT Time (h)** | **Target Vessel Segments** |
| --- | --- | --- | --- | --- | --- | --- | --- | --- |
| Lee et al. 2025 | External Validation | 477 | 23.48% | 60.88 ± 15.87 | 256 (53.67%) | NR | NR | ICA+M1 |
| Rai et al. 2025 | External Validation | 612 | 49.67% | 66.48 ± 16.37 | 296 (48.37%) | 9.28 ± 9.12 | 5.61 ± 8.46 | ICA+M1+M2 |
| Yedavalli et al. 2023 | External Validation | 244 | 47.13% | NR | NR | NR | NR | ICA+M1 |
| Weyland et al. 2022 | External Validation | 154 | 54.55% | 71.36 ± 16.12 | 91 (59.09%) | 14.50 ± 3.33 | NR | ICA+M1+M2 |
| Chung et al. 2025^a^ | External Validation | 65 | 53.85% | NR | NR | NR | NR | ICA+M1+M2 |
| Urra et al. 2025 | External Validation | 189 | 37.57% | NR | NR | NR | NR | ICA+M1+M2 |
| Olive-Gadea et al. 2020 | Internal Validation (Temporal) | 1453 | 56.64% | 70.33 ± 13.33 | 691 (47.56%) | 11.33 ± 9.63 | 2.77 ± 2.04 | Anterior + Posterior (Anterior 95.02%) |
| Kim et al. 2024 | External Validation | 95 | 26.32% | 67.90 ± 13.60 | 56 (58.95%) | 7.93 ± 6.51 | 22.27 ± 40.52 | ICA+M1+M2 |
| Tolhuisen et al. 2020 | External Validation | 103 | 56.31% | NR | NR | NR | NR | ICA+M1+M2 |
| Sunwoo et al. 2026 (US cohort) | External Validation | 240 | 50.00% | 67.80 ± 15.00 | 110 (45.83%) | NR | NR | ICA+M1+M2 |

**Note:** ^a^ Demographic characteristics were reported for the full 534-patient JLK-CTL validation cohort but not separately for the 65-case reader-comparison subset used in the network meta-analysis.

**Abbreviations:** CT, computed tomography; h, hours; ICA, internal carotid artery; LVO, large vessel occlusion; M1, first segment of middle cerebral artery; M2, second segment of middle cerebral artery; NIHSS, National Institutes of Health Stroke Scale; NR, not reported; SD, standard deviation.

## Supplementary Table 9. CT Acquisition Parameters and Image Analysis Methodologies.

| **Study ID** | **AI Software Type** | **Reference Standard Timing** | **Scanner Manufacturer** | **Segmentation** | **Section thickness (mm)** |
| --- | --- | --- | --- | --- | --- |
| Lee et al. 2025 | Commercial | Concurrent (Gap <1h) | Siemens Healthineers (slice: NR) | Auto | 4.00 |
| Rai et al. 2025 | Commercial | Concurrent (Gap <1h) | Siemens, GE Medical Systems, Philips, Toshiba (slice: NR) | Auto | 1.00 |
| Yedavalli et al. 2023 | Commercial | Concurrent (Gap <1h) | Siemens, GE, Philips, Toshiba (slice: NR) | Auto | 5.00 |
| Weyland et al. 2022 | Commercial | Concurrent (Gap <1h) | Siemens Healthineers Somatom/Emotion (16/64-slice); Philips Ingenuity/MX16 (16/64-slice); Toshiba Aquilion (64-slice) | Auto | 1.00 |
| Chung et al. 2025 | Commercial | Concurrent (Gap <1h) | Philips / GE / Siemens / Toshiba (slice: NR) | Auto | 3.00-5.00 |
| Urra et al. 2025 | Commercial | NR | Siemens Healthineers, GE Healthcare, Koninklijke Philips, Canon (slice: NR) | Auto | NR |
| Olive-Gadea et al. 2020 | Commercial | Concurrent (Gap <1h) | Philips, Siemens, GE Medical (slice: NR) | Auto | 3.00-5.00 |
| Kim et al. 2024 | Commercial | Concurrent (Gap <1h) | NR | Auto | 3.00-5.00 |
| Tolhuisen et al. 2020 | laboratory-developed | Concurrent (Gap <1h) | NR | Auto | 2.50 |
| Sunwoo et al. 2026 (US cohort) | Commercial | Within 24h | GE, Siemens, Philips, Canon (slice: NR) | Auto | NR (<2 mm excluded) |

**Abbreviations:** AI, artificial intelligence; CT, computed tomography; GE, General Electric; NR, not reported.

## Supplementary Table 10. Domain-level assessment of model development using PROBAST+AI.

| Author, year | Quality | | | | Applicability concerns | | | Overall judgment | |
| --- | --- | --- | --- | --- | --- | --- | --- | --- | --- |
|  | Participants and data sources^a^ | Predictors^b^ | Outcome^c^ | Analysis^d^ | Participants and data sources^e^ | Predictors^f^ | Outcome^g^ | Quality^h^ | Applicability concerns^i^ |
| Lee et al. 2025 | U | U | U | U | U | U | U | U | U |
| Rai et al. 2025 | U | U | U | U | U | U | U | U | U |
| Yedavalli et al. 2023 | U | U | U | U | U | U | U | U | U |
| Weyland et al. 2022 | U | U | U | U | U | U | U | U | U |
| Chung et al. 2025 | U | U | U | U | U | U | U | U | U |
| Urra et al. 2025 | U | U | U | U | U | U | U | U | U |
| Olive-Gadea et al. 2020 | U | U | U | U | U | U | U | U | U |
| Kim et al. 2024 | L | L | L | U | L | L | L | U | L |
| Tolhuisen et al. 2020 | L | L | L | L | L | L | L | L | L |
| Sunwoo et al. 2026 | U | U | U | U | L | L | L | U | L |

**Note:** Signaling questions are rated as "yes" (Y), "probably yes" (PY), "probably no" (PN), "no" (N), "no information" (NI), and in some cases "not applicable" (NA). All signaling questions are phrased in such a way that "yes" or "probably yes" indicates a low risk of bias. Any signaling questions rated as "no" or "probably no" indicate a potential high risk of bias in that domain. If there are no "no" or "probably no" ratings, but "no information" (NI) is present, the risk of bias in that domain is classified as unclear.

For commercial algorithms, model-development domains were rated as unclear when the validation report did not provide sufficient information on development data sources, preprocessing, model fitting, hyperparameter selection, class-imbalance handling, or data leakage prevention. An unclear judgment reflects incomplete reporting rather than direct evidence of high risk of bias.

a. Participants and data sources

1.1 Were appropriate data sources used?

1.2 Was an appropriate study design used?

1.3 Did the in- and exclusions of study participants result in a representative dataset?

b. Predictors

2.1 Were predictors defined and assessed in a similar way for all participants?

2.2 Was any pre-processing of predictors similar for all participants?

2.3 Were predictor assessments made without knowledge of outcome data?

2.4 Were the predictors included in the model available at the time the model was intended to be used?

c. Outcome

3.1 Were outcomes defined and assessed appropriately?

3.2 Were outcomes defined and assessed in a similar way for all participants?

3.3 Were outcome assessments made without use or knowledge of predictor data?

3.4 Was the time interval between predictor assessment and outcome assessment appropriate?

d. Analysis

4.1 Was there evidence that the sample size was reasonable?

4.2 Were continuous and categorical predictors handled appropriately?

4.3 Were participants with missing or censored data handled appropriately in the analysis?

4.4 If methods to address class imbalance were used, was the model or the model predictions recalibrated?

4.5 Were methods used to address potential model overfitting?

e. Participants and data sources

Concern that the (data of the) included participants do not match the review question or the assessor’s intended use of the prediction model.

f. Predictors

Concern that the definition, pre-processing, assessment, or timing of assessment of the predictors in the model do not match the review question or the assessor’s intended use.

g. Outcome

Concern that the outcome, its definition, assessment, or timing of assessment do not match the review question or the assessor’s intended use.

h. Quality

Low risk: If all four domains were rated low concern regarding quality.

High risk: If at least one domain was rated high concern regarding quality .

Unclear: If at least one domain was rated unclear concern regarding quality and no domains were rated high concern.

i. Applicability concerns

Low risk: If all three domains were rated low concern for applicability.

High risk: If at least one domain was rated high concern for applicability.

Unclear: If at least one domain was rated unclear concern for applicability and no domains were rated high concern.

**Abbreviations:** AI, artificial intelligence; PROBAST, Prediction model Risk Of Bias Assessment Tool, L low; H high; U unclear.

## Supplementary Table 11. Domain-level assessment of model validation using PROBAST+AI.

| Author, year | Risk of bias | | | | Applicability concerns | | | Overall judgment | |
| --- | --- | --- | --- | --- | --- | --- | --- | --- | --- |
|  | Participants and data sources^a^ | Predictors^b^ | Outcome^c^ | Analysis^d^ | Participants and data sources^e^ | Predictors^f^ | Outcome^g^ | Risk of bias^h^ | Applicability concerns ^i^ |
| Lee et al. 2025 | L | L | L | L | L | L | L | L | L |
| Rai et al. 2025 | L | L | L | L | L | L | L | L | L |
| Yedavalli et al. 2023 | L | L | L | L | L | L | L | L | L |
| Weyland et al. 2022 | L | L | L | L | L | L | L | L | L |
| Chung et al. 2025 | L | L | L | L | L | L | L | L | L |
| Urra et al. 2025 | L | L | L | L | L | L | L | L | L |
| Olive-Gadea et al. 2020 | L | L | L | L | L | L | L | L | L |
| Kim et al. 2024 | L | L | L | U | L | L | L | U | L |
| Tolhuisen et al. 2020 | L | L | L | L | L | L | L | L | L |
| Sunwoo et al. 2026 | L | L | L | L | L | L | L | L | L |

**Note:** Signaling questions are rated as "yes" (Y), "probably yes" (PY), "probably no" (PN), "no" (N), "no information" (NI), and in some cases "not applicable" (NA). All signaling questions are phrased in such a way that "yes" or "probably yes" indicates a low risk of bias. Any signaling questions rated as "no" or "probably no" indicate a potential high risk of bias in that domain. If there are no "no" or "probably no" ratings, but "no information" (NI) is present, the risk of bias in that domain is classified as unclear.

a. Participants and data sources

1.1 Were appropriate data sources used?

1.2 Was an appropriate study design used?

1.3 Did the in- and exclusions of study participants result in a representative dataset?

b. Predictors

2.1 Were predictors defined and assessed in a similar way for all participants?

2.2 Was any pre-processing of predictors similar for all participants?

2.3 Were predictor assessments made without knowledge of outcome data?

2.4 Were the predictors included in the model available at the time the model was intended to be used?

c. Outcome

3.1 Were outcomes defined and assessed appropriately?

3.2 Were outcomes defined and assessed in a similar way for all participants?

3.3 Were outcome assessments made without use or knowledge of predictor data?

3.4 Was the time interval between predictor assessment and outcome assessment appropriate?

d. Analysis

4.1 Was model evaluation based on only apparent performance avoided?

4.2 Was there evidence that the sample size was reasonable?

4.3 Were participants with missing or censored data handled appropriately in the analysis?

4.4 If methods to address class imbalance were used, was the evaluation done in a dataset without imbalance correction?

4.5 If data splitting was done to create training and test datasets, was there evidence that data leakage was avoided?

4.6 If resampling methods were used to evaluate model performance, were all model development steps replicated in the resampling process?

4.7 Was the predictive performance of the model evaluated appropriately, e.g., calibration, discrimination, and net benefit?

e. Participants and data sources

Concern that the (data of the) included participants do not match the review question or the assessor’s intended use of the prediction model.

f. Predictors

Concern that the definition, pre-processing, assessment, or timing of assessment of the predictors in the model do not match the review question or the assessor’s intended use.

g. Outcome

Concern that the outcome, its definition, assessment, or timing of assessment do not match the review question or the assessor’s intended use.

h. Risk of bias

Low risk: If all four domains were rated low risk of bias.

High risk: If at least one domain was rated high risk of bias.

Unclear: If at least one domain was rated unclear risk of bias and no domains were rated high risk of bias.

i. Applicability concerns

Low risk: If all three domains were rated low concern for applicability.

High risk: If at least one domain was rated high concern for applicability.

Unclear: If at least one domain was rated unclear concern for applicability and no domains were rated high concern.

**Abbreviations:** AI, artificial intelligence; PROBAST, Prediction model Risk of Bias Assessment Tool, L low; H high; U unclear.

## Supplementary Table 12. QUADAS-3 risk-of-bias and applicability assessment of included diagnostic accuracy studies.

| **Author, year** | | **Risk of bias** | | | | **Applicability concerns** | | | **Overall judgement** | |
| --- | --- | --- | --- | --- | --- | --- | --- | --- | --- | --- |
|  |  | **Participants^a^** | **Index test^b^** | **Target condition^c^** | **Analysis^d^** | **Participants^e^** | **Index test^f^** | **Target condition^g^** | **Risk of bias^h^** | **Applicability concerns^i^** |
| Lee et al. 2025 |  | H | L | H | L | H | L | L | H | H |
| Rai et al. 2025 |  | H | H | L | L | H | L | L | H | H |
| Yedavalli et al. 2023 |  | H | L | L | L | H | L | L | H | H |
| Weyland et al. 2022 |  | H | U | L | L | H | L | L | H | H |
| Chung et al. 2025 |  | H | L | L | L | H | L | L | H | H |
| Urra et al. 2025 |  | H | L | H | L | H | L | L | H | H |
| Olive-Gadea et al. 2020 |  | H | U | L | L | L | L | H | H | H |
| Kim et al. 2024 |  | H | H | L | U | H | L | L | H | H |
| Tolhuisen et al. 2020 |  | H | U | H | L | H | L | L | H | H |
| Sunwoo et al. 2026 (US cohort) |  | H | L | H | L | H | L | H | H | H |

**Note:** Signalling questions are rated as "yes" (Y), "probably yes" (PY), "probably no" (PN), "no" (N), or "no information" (NI). Domain-level judgments are recorded as low, high, or insufficient information. In this table, L indicates low risk/low concern, H indicates high risk/high concern, and U indicates insufficient information/unclear. Participants-domain risk-of-bias judgments explicitly incorporated single-gate design, prospective versus retrospective enrollment, consecutive or random sampling, and representativeness of the intended-use population; Participants applicability was judged separately according to whether the included population, presentation, setting, and prior testing matched the intended emergency-triage use.

a. Participants:

1.1 Was a single-gate design used?

1.2 Were participants prospectively enrolled?

1.3 Was a consecutive or random sample of participants included?

1.4 Is the study group a representative sample of the intended-use population?

b. Index test:

2.1 Was the index test conducted and interpreted according to recommended instructions?

2.2 Were the index test results interpreted without knowledge of the reference standard results?

2.3 Were the index test results interpreted with the same information as would be available in practice?

2.4 If an index test threshold was used, was it standard or pre-specified?

c. Target condition:

3.1 Does the reference standard adequately identify those with and without the target condition?

3.2 Was the target condition assessed in all participants?

3.3 Was the target condition assessed in the same way in all participants?

3.4 Did the reference standard avoid incorporating the index test?

3.5 Was the reference standard conducted and interpreted according to recommended instructions?

3.6 Were the reference standard results interpreted without knowledge of the index test results?

3.7 If a reference standard threshold was used, was it standard or pre-specified?

3.8 Was there an appropriate time interval between index test and reference standard?

d. Analysis:

4.1 Were all participants included in the analysis?

4.2 Were missing data handled appropriately?

4.3 Does the unit of analysis match the ideal test accuracy trial?

4.4 Were the estimates of sensitivity and specificity calculated appropriately?

e. Participants applicability: Concern that the included participants, presentation, setting, or prior testing do not match the review question or intended use of the diagnostic test.

f. Index test applicability: Concern that the index test, technology, execution, interpretation, threshold, or setting does not match the review question or intended use.

g. Target condition applicability: Concern that the target condition definition, reference standard, threshold, or clinical meaning does not match the review question or intended use.

h. Overall risk of bias: Low if all four risk-of-bias domains are low; high if at least one domain is high; unclear/insufficient information if at least one domain is unclear and no domain is high.

i. Overall applicability concerns: Low if all three applicability domains are low; high if at least one domain is high; unclear/insufficient information if at least one domain is unclear and no domain is high.

**Abbreviations:** QUADAS-3, Quality Assessment of Diagnostic Accuracy Studies-3; L, low; H, high; U, insufficient information/unclear.

## Supplementary Table 13. GRADE certainty assessment for pooled sensitivity and specificity estimates.

| **Diagnostic Node** | **Summary accuracy (95% CrI)** | **No. of participants (validation cohorts)** | **Risk of Bias** | **Inconsistency** | **Indirectness** | **Imprecision** | **Publication Bias** | **Effect per 1000 patients tested at 20% pretest probability** | **Final Rating** |
| --- | --- | --- | --- | --- | --- | --- | --- | --- | --- |
| Expert Readers | Sensitivity: 0.62 (0.48 to 0.75) | 1545 participants (7 validation cohorts) | serious | not serious | not serious | serious | not serious | TP: 125 (97 to 151), FN: 75 (49 to 103) | ⊕⊕○○ / Low |
|  | Specificity: 0.86 (0.77 to 0.93) |  | serious | not serious | not serious | serious | not serious | TN: 688 (616 to 740), FP: 112 (60 to 184) | ⊕⊕○○ / Low |
| Non-Expert Readers | Sensitivity: 0.60 (0.45 to 0.75) | 1634 participants (7 validation cohorts) | serious | not serious | not serious | serious | not serious | TP: 121 (90 to 150), FN: 79 (50 to 110) | ⊕⊕○○ / Low |
|  | Specificity: 0.86 (0.75 to 0.93) |  | serious | not serious | not serious | serious | not serious | TN: 687 (600 to 747), FP: 113 (53 to 200) | ⊕⊕○○ / Low |
| Unimodal Imaging AI | Sensitivity: 0.78 (0.68 to 0.86) | 3632 participants (11 validation cohorts) | serious | not serious | not serious | serious | not serious | TP: 156 (136 to 172), FN: 44 (28 to 64) | ⊕⊕○○ / Low |
|  | Specificity: 0.88 (0.81 to 0.94) |  | serious | not serious | not serious | not serious | not serious | TN: 708 (651 to 749), FP: 92 (51 to 149) | ⊕⊕⊕○ / Moderate |
| Clinically-Informed Multimodal AI | Sensitivity: 0.81 (0.64 to 0.92) | 1933 participants (3 validation cohorts) | serious | not serious | not serious | serious | not serious | TP: 162 (128 to 185), FN: 38 (15 to 72) | ⊕⊕○○ / Low |
|  | Specificity: 0.92 (0.80 to 0.98) |  | serious | not serious | not serious | serious | not serious | TN: 734 (638 to 783), FP: 66 (17 to 162) | ⊕⊕○○ / Low |

**Note:** The results in this table should not be interpreted in isolation from the results of the individual included studies contributing to each summary test accuracy measure. These are reported in the main body of the text of the review.

a. Risk of Bias

Risk of bias was informed by validation-stage PROBAST+AI and QUADAS-3 assessments for the studies contributing to each node-specific estimate. PROBAST+AI model-development judgments were not used for GRADE. Downgrading was applied when the contributing evidence showed important risk-of-bias concerns in either complementary framework.

b. Inconsistency

Evaluated by assessing the between-study variance from the bivariate/HSROC model and visual inspection of the summary ROC plot.

Downgraded by 1 level if the 95% prediction region is substantially larger than the 95% confidence region, indicating unexplained wide variance. We did not downgrade for inconsistency in the main analysis because the local node-splitting signal was sparse, limited to a reader-reader sensitivity comparison, and did not drive the primary AI-versus-reader interpretation.

Heterogeneity did not automatically lead to downgrading; downgrading was considered when heterogeneity was substantial, unexplained, and likely to affect clinical interpretation.

c. Indirectness

Evaluated based on the applicability and transferability of the findings to the review question.

Downgrade by 1 level if there are significant concerns that the study populations, index test applications, or reference standards differ substantially from the targeted clinical setting. Outcome Indirectness: Downgrade if inconsistent outcome measures are used, such as estimating sensitivity/specificity indirectly from ROC-based Youden Index.

d. Imprecision

Assessed based on the width of the 95% credible intervals (CrIs) for the summary estimates of test accuracy.

Downgrade by 1 level if the CrIs are excessively wide (e.g., width > 0.20) or if the upper and lower CrI limits cross decision-making thresholds that would lead to different clinical actions (e.g., 0.80).

e. Publication Bias

Evaluated using the Deeks' funnel plot asymmetry test.

Publication bias was evaluated using Deeks' funnel plot asymmetry test and qualitative assessment of selective reporting. Downgrading was applied only when there was clear evidence of asymmetry using a prespecified threshold of P < .05 or compelling qualitative evidence of selective publication.

**Abbreviations:** AI, artificial intelligence; CrI, credible interval; CNN, convolutional neural network; FN, false negative; FP, false positive; GRADE, Grading of Recommendations Assessment, Development and Evaluation; HSROC, hierarchical summary receiver operating characteristic; PROBAST, Prediction model Risk Of Bias Assessment Tool; ROC, receiver operating characteristic; TN, true negative; TP, true positive.

## Supplementary Table 14. GRADE certainty assessment for major comparative diagnostic accuracy estimates.

| **Comparison** | **Outcome** | **Absolute difference (95% CrI)** | **Evidence base** | **Risk of bias** | **Inconsistency** | **Indirectness** | **Imprecision** | **Publication bias** | **Final certainty** |
| --- | --- | --- | --- | --- | --- | --- | --- | --- | --- |
| Unimodal Imaging AI vs Expert Readers | Sensitivity | +0.16 (0.03 to 0.29) | 7 direct datasetsᵃ | Seriousᵃ | Not seriousᶠ | Not serious | Seriousᵍ | Not seriousʰ | Low |
|  | Specificity | +0.02 (-0.06 to 0.12) |  |  |  |  |  |  |  |
| Unimodal Imaging AI vs Non-Expert Readers | Sensitivity | +0.18 (0.03 to 0.32) | 7 direct datasetsᵇ | Seriousᵇ | Not seriousᶠ | Not serious | Seriousᵍ | Not seriousʰ | Low |
|  | Specificity | +0.03 (-0.07 to 0.14) |  |  |  |  |  |  |  |
| Clinically-Informed Multimodal AI vs Expert Readers | Sensitivity | +0.18 (-0.01 to 0.36) | Indirect via Multimodal-Unimodal and Unimodal-Expert evidenceᶜ | Seriousᶜ | Not seriousᶠ | Serious | Seriousᵍ | Not seriousʰ | Very low |
|  | Specificity | +0.06 (-0.08 to 0.17) |  |  |  |  |  |  |  |
| Clinically-Informed Multimodal AI vs Non-Expert Readers | Sensitivity | +0.20 (-0.01 to 0.39) | Indirect via Multimodal-Unimodal and Unimodal-Non-Expert evidenceᵈ | Seriousᵈ | Not seriousᶠ | Serious | Seriousᵍ | Not seriousʰ | Very low |
|  | Specificity | +0.06 (-0.08 to 0.19) |  |  |  |  |  |  |  |
| Clinically-Informed Multimodal AI vs Unimodal Imaging AI | Sensitivity | +0.03 (-0.13 to 0.16) | 3 direct datasetsᵉ | Seriousᵉ | Not seriousᶠ | Not serious | Seriousᵍ | Not seriousʰ | Low |
|  | Specificity | +0.03 (-0.09 to 0.12) |  |  |  |  |  |  |  |

**Note:** Comparative estimates are posterior absolute differences on the probability scale and are presented as the first-listed diagnostic paradigm minus the second-listed paradigm. Risk-of-bias judgments were made separately for each comparison using only validation-stage PROBAST+AI and QUADAS-3 judgments from the studies contributing evidence to that comparison; PROBAST+AI model-development judgments were not used. Sensitivity and specificity were graded separately. Comparative rDORs were considered exploratory and were not separately graded.

a. Unimodal Imaging AI vs Expert Readers: direct evidence from Lee et al. 2025, Rai et al. 2025 Subset A, Yedavalli et al. 2023, Chung et al. 2025, Urra et al. 2025, Tolhuisen et al. 2020, and Sunwoo et al. 2026 (US cohort). Validation-stage PROBAST+AI was low risk in all seven studies. QUADAS-3 overall risk of bias was high in all seven studies; risk of bias was therefore downgraded one level.

b. Unimodal Imaging AI vs Non-Expert Readers: direct evidence from Lee et al. 2025, Rai et al. 2025 Subset A, Yedavalli et al. 2023, Weyland et al. 2022, Urra et al. 2025, Tolhuisen et al. 2020, and Sunwoo et al. 2026 (US cohort). Validation-stage PROBAST+AI was low risk in all seven studies. QUADAS-3 overall risk of bias was high in all seven studies; risk of bias was therefore downgraded one level.

c. Clinically-Informed Multimodal AI vs Expert Readers: no direct head-to-head dataset was available. The indirect comparison was informed by the Multimodal AI vs Unimodal Imaging AI evidence set (Rai et al. 2025 Subset B, Olive-Gadea et al. 2020, and Kim et al. 2024) and the Unimodal Imaging AI vs Expert Readers evidence set listed in footnote a. QUADAS-3 overall risk of bias was high across all contributing studies; risk of bias was judged serious across the constituent evidence sets, and indirectness was downgraded one level because the comparison relied entirely on indirect evidence.

d. Clinically-Informed Multimodal AI vs Non-Expert Readers: no direct head-to-head dataset was available. The indirect comparison was informed by the Multimodal AI vs Unimodal Imaging AI evidence set and the Unimodal Imaging AI vs Non-Expert Readers evidence set listed in footnote b. QUADAS-3 overall risk of bias was high across all contributing studies; risk of bias was judged serious across the constituent evidence sets, and indirectness was downgraded one level because the comparison relied entirely on indirect evidence.

e. Clinically-Informed Multimodal AI vs Unimodal Imaging AI: direct evidence from Rai et al. 2025 Subset B, Olive-Gadea et al. 2020, and Kim et al. 2024. Validation-stage PROBAST+AI was low risk in Rai et al. and Olive-Gadea et al. and unclear in Kim et al.; QUADAS-3 overall risk of bias was high in all three studies. Risk of bias was therefore downgraded one level.

f. Inconsistency was not downgraded for the major AI comparisons because no comparison-specific inconsistency signal was identified. This judgment remains cautious because the sparse network limited the power to detect inconsistency and formal node-splitting was not identifiable for most AI comparisons.

g. Imprecision was downgraded one level for all comparisons because the 95% CrIs were wide and either included the null or, for the two Unimodal Imaging AI sensitivity comparisons, extended from a small to a substantially larger comparative effect.

h. Publication bias was not downgraded because no compelling evidence of publication bias was identified; however, formal assessment was limited by the small evidence base.

**Abbreviations:** AI, artificial intelligence; CrI, credible interval; GRADE, Grading of Recommendations Assessment, Development and Evaluation; PROBAST+AI, Prediction model Risk Of Bias ASsessment Tool for Artificial Intelligence; QUADAS-3, Quality Assessment of Diagnostic Accuracy Studies 3; rDOR, relative diagnostic odds ratio.

## Supplementary Table 15. League table of rDORs.

| **Expert Readers** | 0.92 (0.38 to 2.30) | **2.68 (1.22 to 5.97)** | **5.48 (1.30 to 23.59)** |
| --- | --- | --- | --- |
| 1.09 (0.43 to 2.67) | **Non-Expert Readers** | **2.96 (1.08 to 7.37)** | **6.00 (1.29 to 27.28)** |
| **0.37 (0.17 to 0.82)** | **0.34 (0.14 to 0.92)** | **Unimodal Imaging AI** | 2.04 (0.55 to 7.83) |
| **0.18 (0.04 to 0.77)** | **0.17 (0.04 to 0.77)** | 0.49 (0.13 to 1.81) | **Clinically-Informed Multimodal AI** |

**Note:** Data are presented as posterior medians with 95% CrIs. Comparisons should be read uniformly as the column-defining modality compared to the row-defining modality. Across the entire matrix, an rDOR > 1.00 indicates that the column-defining modality yields higher diagnostic accuracy than the row-defining modality. Comparisons for which the 95% CrI excluded 1.00 are highlighted in bold.

**Abbreviations:** CrI, credible interval; rDOR, relative diagnostic odds ratio.

## Supplementary Table 16. Study-level potential effect modifiers relevant to the transitivity assumption across diagnostic nodes.

| **Study** | **Nodes represented** | **Human-reader expertise** | **Target vessel definition** | **M2 included** | **NCCT section thickness (mm)** | **Reference standard** | **LVO prevalence** | **Validation design** | **AI model / algorithm type** | **Clinical variables in multimodal model** |
| --- | --- | --- | --- | --- | --- | --- | --- | --- | --- | --- |
| Lee et al., 2025 | 1, 2, 3 | Stroke experts >10 years; board-certified specialists and neurology residents; reader-specific specialty mapping partial. | ICA + M1 | No | 4.0 | CTA | 23.48% | External validation | Heuron ELVO; commercial AI | None |
| Rai et al., 2025 | 1, 2, 3, 4  A: 1,2,3;  B: 3,4 | 4 fellowship-trained US board-certified neuroradiologists (≥ 4 years board-certified experience); 6 general radiologists classified as nonexperts. | ICA + M1 + M2 | Yes | 1.0 | CTA | 49.67% | External validation | Brainomix 360 Triage Stroke; commercial AI | NIHSS |
| Yedavalli et al., 2023 | 1, 2, 3 | 3 board-certified neuroradiologists; 8 board-certified general radiologists; experience NR. | ICA + M1 | No | 5.0 | CTA | 47.13% | External validation | RAPID NCCT Stroke; commercial AI | None |
| Weyland et al., 2022 | 2, 3 | 2 blinded neuroradiology residents with 2 and 3 years experience. | ICA + M1 + M2 | Yes | 1.0 | CTA | 54.55% | External validation | Brainomix; commercial AI | None |
| Chung et al., 2025 | 1, 3 | 2 radiologists, each with >10 years NCCT interpretation experience. | ICA + M1 + M2 | Yes | 3.0-5.0 | CTA | 53.85%* | External validation | JLK-CTL; commercial AI | None |
| Urra et al., 2025 | 1, 2, 3 | 2 certified neuroradiologists; 2 general radiologists; experience NR. | ICA + M1 + M2 | Yes | NR | CTA | 37.57% | External validation | Methinks LVO; commercial AI | None |
| Olive-Gadea et al., 2020 | 3, 4 | No eligible human NCCT reader comparator. | Anterior + posterior; 95.02% anterior | Yes | 3.0-5.0 | CTA | 56.64% | Temporal internal validation independent of model development | Methinks LVO / Methinks LVO+; commercial AI | NIHSS; time from symptom onset |
| Kim et al., 2024 | 3, 4 | No eligible human NCCT reader comparator. | ICA + M1 + M2 | Yes | 3.0-5.0 | CTA | 26.32% | External validation | JLK-CTL / JLK-CTL+; commercial AI | NIHSS |
| Tolhuisen et al., 2020 | 1, 2, 3 | Expert neuroradiologist >15 years; neuroradiologist in training >4 years. | ICA + M1 + M2 | Yes | 2.5 | CTA | 56.31% | External validation | 3D CNN; laboratory-developed AI | None |
| Sunwoo et al., 2026 (US cohort) | 1, 2, 3 | 5 US board-certified radiologists: 3 neuroradiologists and 2 general radiologists; experience NR. | ICA + M1 + M2 | Yes | NR† | CTA/MRA/DSA | 50.00% | External validation | JLK-CTL; commercial AI | None |

**Notes:** *For Chung et al., LVO prevalence refers to the 65-case reader-comparison subset used in the network meta-analysis; full-cohort demographics were not reported separately for this subset. †Sunwoo et al. excluded examinations with section thickness <2 mm, but the exact thickness distribution of the included US cohort was not reported. Rai et al. contributed two non-overlapping analytical subsets: Subset A included Nodes 1-3 and Subset B included Nodes 3-4. Commercial/laboratory-developed denotes algorithm origin rather than regulatory status.

**Abbreviations:** AI, artificial intelligence; CTA, computed tomography angiography; DSA, digital subtraction angiography; ICA, internal carotid artery; LVO, large vessel occlusion; M1/M2, first/second segment of the middle cerebral artery; MRA, magnetic resonance angiography; NCCT, noncontrast computed tomography; NIHSS, National Institutes of Health Stroke Scale; NR, not reported. Nodes: 1, Expert Readers; 2, Non-Expert Readers; 3, Unimodal Imaging AI; 4, Clinically-Informed Multimodal AI.

## Supplementary Table 17. Direct and indirect evidence structure and local inconsistency assessment.

| **Comparison** | **Metric** | **Direct validation datasets** | **Independent indirect evidence** | **Direct estimate (95% CrI)** | **Indirect estimate (95% CrI)** | **Inconsistency factor (95% CrI)** |
| --- | --- | --- | --- | --- | --- | --- |
| Non-Expert Readers vs Expert Readers | Sensitivity | 6 | 5 complementary datasets; estimable via Expert Readers → Unimodal Imaging AI → Non-Expert Readers | -0.22 (-0.46 to 0.02) | 1.76 (0.54 to 3.01) | -1.98 (-3.25 to -0.74) |
|  | Specificity |  |  | -0.45 (-0.70 to -0.19) | 0.28 (-1.57 to 2.15) | -0.72 (-2.61 to 1.13) |
| Unimodal Imaging AI vs Expert Readers | Sensitivity | 7 | 4 complementary datasets; not estimable (Expert Readers absent) | 0.91 (0.66 to 1.16) | NE | NE |
|  | Specificity |  |  | 0.49 (0.20 to 0.79) | NE | NE |
| Unimodal Imaging AI vs Non-Expert Readers | Sensitivity | 7 | 4 complementary datasets; not estimable (Non-Expert Readers absent) | 0.95 (0.70 to 1.19) | NE | NE |
|  | Specificity |  |  | 0.87 (0.60 to 1.14) | NE | NE |
| Clinically-Informed Multimodal AI vs Unimodal Imaging AI | Sensitivity | 3 | 8 complementary datasets; not estimable (multimodal AI absent) | 0.00 (-0.22 to 0.22) | NE | NE |
|  | Specificity |  |  | 0.77 (0.51 to 1.03) | NE | NE |

**Note:** Estimates are posterior contrasts on the logit scale. Direct evidence denotes validation datasets containing the corresponding within-study head-to-head comparison. Independent indirect evidence was considered estimable only when the target contrast remained identifiable from the complementary network after separation of its direct evidence. The inconsistency factor was defined as the direct minus indirect contrast and summarized from its posterior distribution with 95% CrIs. NE indicates not estimable because an independent indirect comparison was not identifiable. Comparisons of Clinically-Informed Multimodal AI with either human-reader node had no direct evidence and were therefore indirect-only network comparisons for which node-splitting was not applicable.

**Abbreviations:** AI, artificial intelligence; CrI, credible interval; NE, not estimable.

## Supplementary Table 18. Bayesian meta-regression analysis.

| **Scope** | **Covariates** | **Categories** | **Studies (Validation sets)^a^** | **Sensitivity β (95% CrI)^b^** | **Specificity β (95% CrI)^b^** |
| --- | --- | --- | --- | --- | --- |
| Global | CT Slice Thickness | <3 mm | 3 (4) | Reference | Reference |
|  |  | ≥3 mm | 5 (5) | -0.12 (-1.37 to 1.12) | -0.29 (-1.60 to 1.01) |
|  | M2 Segment Inclusion | Proximal only (ICA+M1) | 2 (2) | Reference | Reference |
|  |  | Included (+M2) | 8 (9) | 0.08 (-1.16 to 1.31) | -0.18 (-1.41 to 1.04) |
| AI-restricted | Validation design | Internal | 1 (1) | Reference | Reference |
|  |  | External | 9 (10) | -0.26 (-1.87 to 1.38) | 1.29 (-0.10 to 2.68) |
|  | Algorithm Origin | laboratory-developed | 1 (1) | Reference | Reference |
|  |  | Commercial | 9 (10) | 0.16 (-1.83 to 1.95) | 1.18 (-0.95 to 3.27) |

**Note:** ^a^ Studies are counted as independent original studies, with validation sets shown in parentheses; Rai et al. (2025) Subsets A and B were treated as two validation sets from one original study. ^b^ Regression coefficients are posterior means on the logit scale with 95% credible intervals; the category labeled Reference served as the reference level. AI-restricted covariates were modeled only for AI nodes. The algorithm-origin comparison should be interpreted as exploratory because only one laboratory-developed AI row was available.

**Abbreviations:** AI, artificial intelligence; CrI, credible interval; CT, computed tomography; ICA, internal carotid artery; M1, first segment of the middle cerebral artery; M2, second segment of middle cerebral artery.

## Supplementary Table 19. Sensitivity analyses.

| **Diagnostic Node** | **Metrics** | **Overall Network** | **SUCRA Rank** | **External Validation Only** | **SUCRA Rank** | **Commercial AI software** | **SUCRA Rank** | **Including M2** | **SUCRA Rank** | **Removing Rai 2025 Subset A** | **SUCRA Rank** | **Excluding mixed or non-concurrent reference standards** | **SUCRA Rank** | **Half-normal(0,1) heterogeneity prior** | **SUCRA Rank** |
| --- | --- | --- | --- | --- | --- | --- | --- | --- | --- | --- | --- | --- | --- | --- | --- |
| Expert Readers | Sensitivity | 0.62 (0.48 to 0.75) | #3 (21.77%) | 0.63 (0.47 to 0.76) | #3 (23.25%) | 0.57 (0.43 to 0.70) | #4 (13.41%) | 0.62 (0.41 to 0.80) | #4 (20.08%) | 0.64 (0.48 to 0.79) | #3 (22.25%) | 0.65 (0.48 to 0.79) | #3 (26.40%) | 0.62 (0.49 to 0.75) | #3 (21.74%) |
|  | Specificity | 0.86 (0.77 to 0.93) | #4 (30.89%) | 0.86 (0.78 to 0.92) | #4 (24.68%) | 0.88 (0.80 to 0.94) | #3 (43.70%) | 0.87 (0.76 to 0.94) | #3 (35.47%) | 0.84 (0.73 to 0.92) | #4 (21.00%) | 0.86 (0.75 to 0.93) | #4 (31.34%) | 0.86 (0.77 to 0.92) | #4 (30.50%) |
|  | DOR | 11.53 (5.15 to 22.48) | #3 (20.03%) | 11.87 (5.49 to 22.72) | #3 (19.45%) | 11.11 (4.75 to 22.51) | #3 (22.50%) | 13.31 (4.58 to 31.42) | #4 (19.42%) | 10.77 (4.28 to 22.59) | #4 (14.48%) | 13.04 (4.98 to 28.07) | #3 (21.45%) | 11.41 (5.29 to 21.79) | #3 (20.09%) |
| Non-Expert Readers | Sensitivity | 0.60 (0.45 to 0.75) | #4 (14.12%) | 0.61 (0.44 to 0.76) | #4 (16.17%) | 0.59 (0.44 to 0.75) | #3 (21.53%) | 0.64 (0.41 to 0.83) | #3 (27.39%) | 0.63 (0.45 to 0.79) | #4 (16.51%) | 0.60 (0.42 to 0.77) | #4 (11.79%) | 0.60 (0.45 to 0.74) | #4 (13.91%) |
|  | Specificity | 0.86 (0.75 to 0.93) | #3 (31.65%) | 0.86 (0.76 to 0.93) | #3 (27.46%) | 0.85 (0.73 to 0.93) | #4 (17.86%) | 0.86 (0.72 to 0.94) | #4 (27.08%) | 0.86 (0.74 to 0.94) | #3 (39.31%) | 0.87 (0.74 to 0.95) | #3 (39.64%) | 0.86 (0.75 to 0.93) | #3 (30.91%) |
|  | DOR | 10.94 (4.09 to 24.08) | #4 (14.98%) | 11.57 (4.33 to 25.61) | #4 (15.58%) | 9.66 (3.36 to 22.72) | #4 (12.33%) | 13.93 (3.77 to 37.21) | #3 (19.81%) | 12.71 (4.02 to 31.00) | #3 (21.88%) | 12.26 (3.80 to 30.15) | #4 (16.17%) | 10.72 (4.23 to 22.94) | #4 (14.51%) |
| Unimodal Imaging AI | Sensitivity | 0.78 (0.68 to 0.86) | #2 (76.34%) | 0.78 (0.66 to 0.87) | #2 (76.85%) | 0.77 (0.67 to 0.85) | #2 (76.24%) | 0.77 (0.61 to 0.89) | #2 (72.52%) | 0.79 (0.68 to 0.88) | #2 (76.03%) | 0.78 (0.66 to 0.87) | #2 (75.53%) | 0.78 (0.68 to 0.86) | #2 (76.29%) |
|  | Specificity | 0.88 (0.81 to 0.94) | #2 (54.13%) | 0.90 (0.84 to 0.95) | #2 (62.59%) | 0.89 (0.83 to 0.94) | #2 (55.77%) | 0.89 (0.81 to 0.95) | #2 (53.33%) | 0.88 (0.80 to 0.94) | #2 (54.98%) | 0.88 (0.79 to 0.94) | #2 (48.19%) | 0.89 (0.82 to 0.93) | #2 (54.91%) |
|  | DOR | 30.89 (14.19 to 60.27) | #2 (70.20%) | 37.28 (16.43 to 74.33) | #2 (71.70%) | 32.45 (14.54 to 64.57) | #2 (71.09%) | 34.21 (12.05 to 77.52) | #2 (68.40%) | 31.96 (13.39 to 67.51) | #2 (69.48%) | 30.04 (12.27 to 63.46) | #2 (68.57%) | 30.72 (14.71 to 58.32) | #2 (70.43%) |
| Clinically-Informed Multimodal AI | Sensitivity | 0.81 (0.64 to 0.92) | **#1 (87.76%)** | 0.80 (0.56 to 0.95) | **#1 (83.73%)** | 0.80 (0.64 to 0.92) | **#1 (88.83%)** | 0.80 (0.50 to 0.96) | **#1 (80.00%)** | 0.81 (0.62 to 0.93) | **#1 (85.20%)** | 0.81 (0.62 to 0.93) | **#1 (86.28%)** | 0.81 (0.65 to 0.92) | **#1 (88.06%)** |
|  | Specificity | 0.92 (0.80 to 0.98) | **#1 (83.32%)** | 0.93 (0.80 to 0.99) | **#1 (85.28%)** | 0.92 (0.81 to 0.98) | **#1 (82.68%)** | 0.93 (0.78 to 0.99) | **#1 (84.12%)** | 0.92 (0.79 to 0.98) | **#1 (84.72%)** | 0.92 (0.78 to 0.98) | **#1 (80.83%)** | 0.92 (0.80 to 0.98) | **#1 (83.67%)** |
|  | DOR | 75.42 (15.63 to 235.86) | **#1 (94.79%)** | 121.67 (14.03 to 478.06) | **#1 (93.27%)** | 75.40 (15.19 to 237.97) | **#1 (94.08%)** | 133.08 (11.45 to 585.02) | **#1 (92.38%)** | 84.09 (14.29 to 282.02) | **#1 (94.15%)** | 78.70 (13.57 to 264.46) | **#1 (93.81%)** | 72.67 (16.08 to 216.42) | **#1 (94.98%)** |

**Note:** Across all six sensitivity analyses, both AI diagnostic nodes remained the top two DOR-ranked nodes. SA-1 to SA-5 addressed validation design, anterior-circulation restriction, algorithm origin, M2 inclusion, dual-subset handling, and mixed or non-concurrent reference-standard characteristics. SA-6 used an alternative half-normal(0,1) heterogeneity prior and produced no material change in node-level sensitivity, specificity, DOR estimates, or the exploratory diagnostic hierarchy. These sensitivity analyses should be interpreted as robustness checks rather than independent confirmatory analyses.

**Abbreviations:** AI, artificial intelligence; CrI, credible interval; DOR, diagnostic odds ratio; NCCT, noncontrast computed tomography; SUCRA, surface under the cumulative ranking curve.

## Supplementary Table 20. Absolute diagnostic outcomes per 1000 patients across clinically relevant LVO prevalence scenarios.

| **Pretest probability** | **Diagnostic node** | **True positives per 1000 (95% CrI)** | **False negatives per 1000 (95% CrI)** | **False positives per 1000 (95% CrI)** | **True negatives per 1000 (95% CrI)** |
| --- | --- | --- | --- | --- | --- |
| 20% (suspected AIS / emergency triage) | Expert Readers | 125 (97 to 151) | 75 (49 to 103) | 112 (60 to 184) | 688 (616 to 740) |
|  | Non-Expert Readers | 121 (90 to 150) | 79 (50 to 110) | 113 (53 to 200) | 687 (600 to 747) |
|  | Unimodal Imaging AI | 156 (136 to 172) | 44 (28 to 64) | 92 (51 to 149) | 708 (651 to 749) |
|  | Clinically-Informed Multimodal AI | 162 (128 to 185) | 38 (15 to 72) | 66 (17 to 162) | 734 (638 to 783) |
| 30% (confirmed / high-suspicion AIS) | Expert Readers | 187 (145 to 226) | 113 (74 to 155) | 98 (53 to 161) | 602 (539 to 647) |
|  | Non-Expert Readers | 181 (135 to 225) | 119 (75 to 165) | 99 (46 to 175) | 601 (525 to 654) |
|  | Unimodal Imaging AI | 234 (204 to 258) | 66 (42 to 96) | 81 (45 to 131) | 619 (569 to 655) |
|  | Clinically-Informed Multimodal AI | 243 (192 to 277) | 57 (23 to 108) | 57 (15 to 142) | 643 (558 to 685) |
| 49.8% (enriched validation-cohort median) | Expert Readers | 311 (241 to 375) | 187 (123 to 257) | 70 (38 to 116) | 432 (386 to 464) |
|  | Non-Expert Readers | 301 (225 to 374) | 197 (124 to 273) | 71 (33 to 126) | 431 (376 to 469) |
|  | Unimodal Imaging AI | 389 (339 to 429) | 109 (69 to 159) | 58 (32 to 94) | 444 (408 to 470) |
|  | Clinically-Informed Multimodal AI | 403 (319 to 460) | 95 (38 to 179) | 41 (11 to 102) | 461 (400 to 491) |

**Note:** Absolute outcomes are presented as posterior expected counts per 1000 patients with 95% credible intervals (CrIs). For each prevalence scenario, LVO-positive and LVO-negative counts were fixed according to the specified pretest probability, and true-positive, false-negative, false-positive, and true-negative counts were derived from the posterior sensitivity and specificity distributions for each diagnostic node. The 20% scenario is the primary illustration for suspected AIS or emergency triage populations; 30% represents confirmed or high-suspicion AIS populations; and 49.8% represents the enriched median LVO prevalence of the included validation cohorts.

**Abbreviations:** AI, artificial intelligence; AIS, acute ischemic stroke; CrI, credible interval; LVO, large vessel occlusion.

## Supplementary Figure 1. Forest plots of sensitivity, specificity, and diagnostic odds ratio for individual studies in Node 1 (Expert Readers).

Forest plots illustrating the study-level estimates of sensitivity, specificity, and diagnostic odds ratio (DOR) for primary studies categorized under Node 1 (Expert Readers). Data are presented as individual point estimates with corresponding 95% CIs based on a frequentist framework. DOR is displayed on a logarithmic scale. These plots visualize the raw diagnostic accuracy distribution and intra-node heterogeneity prior to network synthesis. No pooled summary estimates are shown; node-level pooled results derived from the Bayesian network meta-analysis are reported in the main text.

Abbreviations: CI, confidence interval; DOR, diagnostic odds ratio.

## Supplementary Figure 2. Forest plots of sensitivity, specificity, and diagnostic odds ratio for individual studies in Node 2 (Non-Expert Readers).

Forest plots illustrating the study-level estimates of sensitivity, specificity, and diagnostic odds ratio (DOR) for primary studies categorized under Node 2 (Non-Expert Readers). Data are presented as individual point estimates with corresponding 95% CIs based on a frequentist framework. DOR is displayed on a logarithmic scale. These plots visualize the raw diagnostic accuracy distribution and intra-node heterogeneity prior to network synthesis. No pooled summary estimates are shown; node-level pooled results derived from the Bayesian network meta-analysis are reported in the main text.

Abbreviations: CI, confidence interval; DOR, diagnostic odds ratio.

## Supplementary Figure 3. Forest plots of sensitivity, specificity, and diagnostic odds ratio for individual studies in Node 3 (Unimodal Imaging AI).

Forest plots illustrating the study-level estimates of sensitivity, specificity, and diagnostic odds ratio (DOR) for primary studies categorized under Node 3 (Unimodal Imaging AI; non-contrast CT input only). Data are presented as individual point estimates with corresponding 95% CIs based on a frequentist framework. DOR is displayed on a logarithmic scale. These plots visualize the raw diagnostic accuracy distribution and intra-node heterogeneity prior to network synthesis. No pooled summary estimates are shown; node-level pooled results derived from the Bayesian network meta-analysis are reported in the main text.

Abbreviations: AI, artificial intelligence; CI, confidence interval; DOR, diagnostic odds ratio.

## Supplementary Figure 4. Forest plots of sensitivity, specificity, and diagnostic odds ratio for individual studies in Node 4 (Clinically-Informed Multimodal AI).

Forest plots illustrating the study-level estimates of sensitivity, specificity, and diagnostic odds ratio (DOR) for primary studies categorized under Node 4 (Clinically-Informed Multimodal AI; non-contrast CT combined with clinical variables including NIHSS). Data are presented as individual point estimates with corresponding 95% CIs based on a frequentist framework. DOR is displayed on a logarithmic scale. These plots visualize the raw diagnostic accuracy distribution and intra-node heterogeneity prior to network synthesis. No pooled summary estimates are shown; node-level pooled results derived from the Bayesian network meta-analysis are reported in the main text.

Abbreviations: AI, artificial intelligence; CI, confidence interval; DOR, diagnostic odds ratio; NIHSS, National Institutes of Health Stroke Scale.

## Supplementary Figure 5. Markov chain Monte Carlo trace plots for convergence diagnostics of the Bayesian network meta-analysis model.

Trace plots of posterior samples for pooled sensitivity, specificity, and diagnostic odds ratio (DOR) across all four nodes: Expert Readers (Node 1), Non-Expert Readers (Node 2), Unimodal Imaging AI (Node 3), and Clinically-Informed Multimodal AI (Node 4). Each panel displays the 20,000 retained post-burn-in draws from each of four parallel chains, yielding 80,000 retained posterior draws across chains. Adequate convergence is indicated by well-mixed, stationary chains with no discernible trends or persistent separation between chains. The x-axis represents the retained iteration number; the y-axis represents the sampled parameter value on the probability scale for sensitivity and specificity and on the natural scale for DOR.

Abbreviations: AI, artificial intelligence; DOR, diagnostic odds ratio; MCMC, Markov chain Monte Carlo.

## Supplementary Figure 6. Markov chain Monte Carlo posterior density overlays for convergence diagnostics of the Bayesian network meta-analysis model.

Kernel density plots of posterior samples for pooled sensitivity, specificity, and diagnostic odds ratio (DOR) across all four nodes: Expert Readers (Node 1), Non-Expert Readers (Node 2), Unimodal Imaging AI (Node 3), and Clinically-Informed Multimodal AI (Node 4). Each panel displays the posterior density estimates from four parallel chains (displayed in varying shades of blue). Adequate convergence is indicated by substantial overlap of density curves across chains. The x-axis represents the parameter value on the probability scale for sensitivity and specificity and on the natural scale for DOR; the y-axis represents the posterior density.

Abbreviations: AI, artificial intelligence; DOR, diagnostic odds ratio; MCMC, Markov chain Monte Carlo.

## Supplementary Figure 7. Design-by-treatment interaction heatmap for assessment of statistical inconsistency in the Bayesian network meta-analysis.

ΔDIC was calculated as DICinconsistency − DICconsistency. The observed ΔDIC of −0.19 indicates negligible separation in relative model fit and should not be interpreted as evidence for the absence of inconsistency. Interpretation is limited by the sparse evidence network. Study-design labels denote combinations of diagnostic nodes: A, Expert Readers; B, Non-Expert Readers; C, Unimodal Imaging AI; D, Clinically-Informed Multimodal AI.

Abbreviations: AI, artificial intelligence; DIC, deviance information criterion.

## Supplementary Figure 8. Node-splitting assessment of local inconsistency in the Bayesian diagnostic test accuracy network meta-analysis.

Direct evidence was compared with the complementary network only when an independently identifiable indirect estimate was available. Formal node-splitting was identifiable for the Non-Expert Readers versus Expert Readers comparison, with contrasts defined as Non-Expert Readers minus Expert Readers. For sensitivity, the direct and indirect contrasts were −0.22 (95% CrI, −0.46 to 0.02) and 1.76 (95% CrI, 0.54 to 3.01), respectively; for specificity, they were −0.45 (−0.70 to −0.19) and 0.28 (−1.57 to 2.15). Independent indirect estimates were not identifiable for the remaining observed edges; only their direct estimates are shown. Posterior inconsistency factors and the direct/indirect evidence structure are provided in Supplementary Table 17.

Abbreviations: AI, artificial intelligence; CrI, credible interval; MA, meta-analysis; NMA, network meta-analysis.

## Supplementary Figure 9. QUADAS-3 risk-of-bias and applicability judgments.

QUADAS-3 judgments are shown for risk-of-bias and applicability domains. L, low risk/low concern; U, unclear or insufficient information; H, high risk/high concern; App, applicability; RoB, risk of bias; QUADAS-3, Quality Assessment of Diagnostic Accuracy Studies 3.

## Supplementary Figure 10. Posterior density distributions of pooled sensitivity and specificity from the Bayesian network meta-analysis main analysis.

Ridge plots displaying the posterior probability density distributions of pooled sensitivity (left panel) and specificity (right panel) for each of the four diagnostic nodes: Expert Readers (blue), Non-Expert Readers (teal), Unimodal Imaging AI (yellow), and Clinically-Informed Multimodal AI (pink). The x-axis represents the estimated probability on the probability scale. The width and shape of each distribution reflect the posterior uncertainty around the pooled estimate; narrower distributions indicate greater precision. A rightward shift in the distribution indicates higher diagnostic accuracy for the corresponding parameter.

Abbreviations: AI, artificial intelligence.

## Supplementary Figure 11. Pooled sensitivity and specificity from the Bayesian network meta-analysis main analysis.

Forest plot displaying the pooled posterior mean estimates of sensitivity (left panel) and specificity (right panel) with corresponding 95% credible intervals (CrI) for each of the four diagnostic nodes: Expert Readers (blue), Non-Expert Readers (teal), Unimodal Imaging AI (yellow), and Clinically-Informed Multimodal AI (pink). Estimates were derived from the bivariate binomial-normal model with baseline-contrast parameterization fitted via Markov chain Monte Carlo sampling. The x-axis represents the probability scale.

Abbreviations: AI, artificial intelligence; CrI, credible interval.

## Supplementary Figure 12. Pooled diagnostic odds ratio from the Bayesian network meta-analysis main analysis.

Forest plot displaying the pooled posterior mean estimates of the diagnostic odds ratio (DOR) with corresponding 95% credible intervals (CrI) for each of the four diagnostic nodes: Expert Readers (blue), Non-Expert Readers (teal), Unimodal Imaging AI (yellow), and Clinically-Informed Multimodal AI (pink). Estimates were derived from the bivariate binomial-normal model with baseline-contrast parameterization fitted via Markov chain Monte Carlo sampling. DOR is displayed on a logarithmic scale; the dashed vertical line at 1 denotes no discriminative ability.

Abbreviations: AI, artificial intelligence; CrI, credible interval; DOR, diagnostic odds ratio.

## Supplementary Figure 13. Node-specific Fagan nomograms at a pretest probability of 20% for suspected AIS or emergency department triage populations.

Solid lines represent positive likelihood ratios (rule-in capacity); dashed lines represent negative likelihood ratios (rule-out capacity). This scenario approximates suspected AIS or emergency department triage populations before CTA confirmation.

Abbreviations: AIS, acute ischemic stroke; CTA, computed tomography angiography; LR, likelihood ratio.

## Supplementary Figure 14. Node-specific Fagan nomograms at a pretest probability of 30% for confirmed or high-suspicion AIS populations.

Solid lines represent positive likelihood ratios (rule-in capacity); dashed lines represent negative likelihood ratios (rule-out capacity). This scenario approximates confirmed AIS or high-suspicion stroke populations.

Abbreviations: AIS, acute ischemic stroke; LR, likelihood ratio.

## Supplementary Figure 15. Node-specific Fagan nomograms at a pretest probability of 49.8% (median LVO prevalence across included validation cohorts).

Solid lines represent positive likelihood ratios (rule-in capacity); dashed lines represent negative likelihood ratios (rule-out capacity).

Abbreviations: LR, likelihood ratio; LVO, large vessel occlusion.

## Supplementary Figure 16. Deeks' funnel plot asymmetry test for assessment of publication bias.

Funnel plot displaying the log diagnostic odds ratio (logDOR) on the x-axis against the inverse square root of the effective sample size on the y-axis for all study-level observations across the four diagnostic nodes: Expert Readers (blue), Non-Expert Readers (teal), Unimodal Imaging AI (yellow), and Clinically-Informed Multimodal AI (pink). Point size is proportional to sample size. The dashed regression line represents Deeks' linear regression of logDOR on the inverse square root of effective sample size. No clear funnel-plot asymmetry was detected (Deeks' test *P* = 0.76), but this assessment should be interpreted cautiously because of the small evidence base.

Abbreviations: AI, artificial intelligence; DOR, diagnostic odds ratio.

**References**

Full bibliographic details for the studies, reporting guidelines, and named methods cited in these supplementary materials are provided below.

1. Salameh JP, Bossuyt PM, McGrath TA, et al. Preferred reporting items for systematic review and meta-analysis of diagnostic test accuracy studies (PRISMA-DTA): explanation, elaboration, and checklist. BMJ. 2020;370:m2632. doi:10.1136/bmj.m2632.

2. Hutton B, Salanti G, Caldwell DM, et al. The PRISMA extension statement for reporting of systematic reviews incorporating network meta-analyses of health care interventions: checklist and explanations. Ann Intern Med. 2015;162(11):777-784. doi:10.7326/M14-2385. PMID:26030634

3. Page MJ, McKenzie JE, Bossuyt PM, et al. The PRISMA 2020 statement: an updated guideline for reporting systematic reviews. BMJ. 2021;372:n71. doi:10.1136/bmj.n71. PMID:33782057

4. Rethlefsen ML, Kirtley S, Waffenschmidt S, et al. PRISMA-S: an extension to the PRISMA Statement for Reporting Literature Searches in Systematic Reviews. Syst Rev. 2021;10(1):39. doi:10.1186/s13643-020-01542-z

5. Sanders JV, Keigher K, Oliver M, Joshi K, Lopes D. Methinks AI software for identifying large vessel occlusion in non-contrast head CT: A pilot retrospective study in American population. Interv Neuroradiol. Published online July 25, 2025. doi:10.1177/15910199251362073.

6. Fussell DA, Lopez JL, Chang PD. A Deep Learning Model to Detect Acute MCA Occlusion on High-Resolution Noncontrast Head CT. AJNR Am J Neuroradiol. 2026;47(2):386-393. doi:10.3174/ajnr.A8954.

7. You J, Tsang ACO, Yu PLH, Tsui ELH, Woo PPS, Lui CSM, Leung GKK. Automated Hierarchy Evaluation System of Large Vessel Occlusion in Acute Ischemia Stroke. Front Neuroinform. 2020;14:13. doi:10.3389/fninf.2020.00013.

8. Lee SJ, Kim D, Choi DH, et al. Using a Deep Learning-Based Decision Support System to Predict Emergent Large Vessel Occlusion Using Non-Contrast Computed Tomography. J Clin Med. 2025;14(13):4635. doi:10.3390/jcm14134635

9. Rai AT, Al Halak A, Abdalkader M, et al. Artificial Intelligence–Driven Detection of Large Vessel Occlusions on NCCT: A Multi-Institutional Study. Am J Neuroradiol. 2025;46(12):2528-2534. doi:10.3174/ajnr.A8923

10. Yedavalli V, Heit JJ, Dehkharghani S, et al. Performance of RAPID noncontrast CT stroke platform in large vessel occlusion and intracranial hemorrhage detection. Front Neurol. 2023;14:1324088. doi:10.3389/fneur.2023.1324088

11. Weyland CS, Papanagiotou P, Schmitt N, et al. Hyperdense Artery Sign in Patients With Acute Ischemic Stroke–Automated Detection With Artificial Intelligence-Driven Software. Front Neurol. 2022;13:807145. doi:10.3389/fneur.2022.807145

12. Chung J, Lee M, Ha SY, et al. Multicenter Validation of Artificial Intelligence Predicting Anterior Circulation Large Vessel Occlusion Using Noncontrast Head CT. Stroke Vasc Interv Neurol. 2025;5(5):e001788. doi:10.1161/SVIN.125.001788

13. Urra X, Rai A, Hernandez M, et al. Evaluation of a Deep Learning Tool for Detecting Large Vessel Occlusion and Intracranial Hemorrhage on Noncontrast Computed Tomography Scans. Stroke Vasc Interv Neurol. 2025;5(6):e001872. doi:10.1161/SVIN.125.001872

14. Sunwoo L, Ryu WS, Buch K, et al. AI assisted detection of large vessel occlusion on non-contrast CT: multinational validation and reader study. J NeuroInterventional Surg. Published online May 22, 2026:jnis-2026-025339. doi:10.1136/jnis-2026-025339

15. Olive-Gadea M, Crespo C, Granes C, et al. Deep Learning Based Software to Identify Large Vessel Occlusion on Noncontrast Computed Tomography. Stroke. 2020;51(10):3133-3137. doi:10.1161/STROKEAHA.120.030326

16. Kim PE, Yang H, Kim D, et al. Automated Prediction of Proximal Middle Cerebral Artery Occlusions in Noncontrast Brain Computed Tomography. Stroke. 2024;55(6):1609-1618. doi:10.1161/STROKEAHA.123.045772

17. Tolhuisen ML, Ponomareva E, Boers AMM, et al. A Convolutional Neural Network for Anterior Intra-Arterial Thrombus Detection and Segmentation on Non-Contrast Computed Tomography of Patients with Acute Ischemic Stroke. Appl Sci. 2020;10(14):4861. doi:10.3390/app10144861

18. Moons KGM, Damen JAA, Kaul T, et al. PROBAST+AI: an updated quality, risk of bias, and applicability assessment tool for prediction models using regression or artificial intelligence methods. BMJ. 2025;388:e082505. doi:10.1136/bmj-2024-082505

19. Whiting PF, Tomlinson E, Rutjes AWS, et al. QUADAS-3: A Revised Tool for the Quality Assessment of Diagnostic Test Accuracy Studies. Ann Intern Med. 2026;179(4):548-555. doi:10.7326/ANNALS-25-02104

20. Gopalakrishna G, Mustafa RA, Davenport C, et al. Applying Grading of Recommendations Assessment, Development and Evaluation (GRADE) to diagnostic tests was challenging but doable. J Clin Epidemiol. 2014;67(7):760-768. doi:10.1016/j.jclinepi.2014.01.006. PMID:24725643

21. Fagan TJ. Letter: Nomogram for Bayes’s theorem. N Engl J Med. 1975;293(5):257. doi:10.1056/NEJM197507312930513. PMID:1143310

22. Deeks JJ, Macaskill P, Irwig L. The performance of tests of publication bias and other sample size effects in systematic reviews of diagnostic test accuracy was assessed. J Clin Epidemiol. 2005;58(9):882-893. doi:10.1016/j.jclinepi.2005.01.016. PMID:16085191
